# An assessment of the khat and vegetable trade in the local economic ecosystem: The case of northern Madagascar

**DOI:** 10.1371/journal.pone.0331722

**Published:** 2026-06-11

**Authors:** Candicia M. Kotra, Lisa L. Gezon

**Affiliations:** 1 Department of Management, University of Antsiranana, Antsiranana, Madagascar; 2 Department of Anthropology, University of Alabama at Birmingham, Birmingham, Alabama, United States of America; PLOS ONE, UNITED KINGDOM OF GREAT BRITAIN AND NORTHERN IRELAND

## Abstract

**Background:**

Khat is widely grown and consumed in parts of Africa. Its trade supports the local economy through income generation and tax contributions. This research explores the economic impact of khat production and trade in northern Madagascar by comparing its income with that of the vegetable trade.

**Methods:**

We compared the profitability of khat and vegetable trading using a mixed-methods approach. Qualitative data were collected through interviews with nine city managers from two major khat-producing municipalities and with two key khat producers in the city of Diego-Suarez. Participant observation was also conducted at sales points across the city. Quantitative data were collected to twenty sellers to complement and validate the qualitative findings. Khat and vegetable sellers maintained daily income diaries to record variables such as income and purchase prices. Data were collected using KoboToolbox and entered via KoboCollect. In addition, to analyze longer-term financial trends, we examined five years of municipal financial statements from the two main production areas, Joffre-ville and Antsalaka..

**Findings:**

The results confirm our hypothesis that khat boosts local economic development by generating employment and municipal taxes that support infrastructure and local development. Further, khat selling proved to be more profitable than vegetable selling, with higher overall earnings despite seasonal price fluctuations. We found that vendors with family ties to khat farmers earn considerably more than others. Vegetables remain attractive to vendors who lack social contacts or capital to invest in khat, or who desire more market stability.

**Discussion and significance:**

Overall, our findings confirm that khat plays a central and complementary role in the local economic ecosystem. Despite its controversial status as a psychoactive crop that is illegal in much of the world, we argue that it should be supported and regulated like other psychoactive crops such as tobacco, tea and coffee.

**Limitations:**

Limitations include a small sample size, the complexity of the phenomenon, and data collection limited to a single year. Limitations were mitigated through in-depth qualitative research, and triangulation through informal interviews and field observations.

## Introduction

Khat, also known as *qat* in Yemen [1–4] and miraa in Kenya [5] is known scientifically as *Catha edulis* [6–11]. Khat is a bushy plant whose leaves produce a mild stimulant effect when chewed [5,12]. Khat is widely consumed in countries across the Horn of Africa, the Middle East, and beyond [2,6–8,13,14]. Among East African countries, Ethiopia and Kenya are the two leading producers and exporters of khat [13].

In Madagascar, khat consumption has increased notably since the 1990s. Its introduction to the country came through the city of Antsiranana (Diego-Suarez) and dates to the early 20^th^ century, when it was brought by Yemeni dock workers during the French colonial period [2,13,15]. Nowadays, khat consumption has spread to urban centers throughout the country, but it is concentrated in the North. The two main areas of production are the towns of Joffre-ville and Antsalaka, while the city and regional capital of Diego-Suarez functions as a major trade hub. This expansion mirrors global trends, as khat has become a commercial crop in other countries [10,16], earning the label such as “green gold” in Kenya and Madagascar [5,15]. For many consumers and scholars, khat is not viewed as a dangerous drug but rather as a socially acceptable product, akin to other legal psychoactive substances such as tobacco and coffee [17,18].

Historically, the fertile highland areas around Madagascar’s Amber Mountain, including Joffre-ville and Antsalaka, were known for vegetable farming during the French colonial era. However, recent years have seen a shift from food crop cultivation toward khat, which has raised concerns among local authorities regarding food security and land use [13]. This change has prompted local stakeholders to seek data on the economic effects of khat to inform governance strategies and policymaking at both municipal and regional levels [13]. In order to understand the relative place of khat in the local economy, this research provides a comparative study of khat, a highly profitable crop, and vegetables, which are vital for food security but have been increasingly abandoned in favor of khat cultivation. While this shift poses a risk to food security in northern Madagascar, we propose that a balance between the two could be key to local development for municipalities.

### Study purpose and hypotheses

Our overall goal has been to understand how and why khat is an important part of the economy both by studying its direct economic impacts and by examining its earnings in comparison with another prominent local perishable commodity: vegetables.

Hypothesis 1: Khat-related employment and earnings from municipal taxation result in local economic development.
Hypothesis 2A: Khat sellers earn more than vegetable sellers.
Hypothesis 2B: Khat earnings are higher in the rainy season than in the dry season

To verify those hypotheses, we adopted a mixed-methods approach to examine key aspects of the khat industry. Qualitative data were collected through interviews with city managers from two major khat-producing municipalities, providing insights into local governance and economic factors. Quantitative data were gathered from daily diaries maintained by khat and vegetable sellers, tracking variables such as income and purchase prices. We also conducted a financial analysis in the two producer municipalities of Antsalaka and Joffre-ville to analyse the tax revenue trends and impacts.

We hypothesized that khat-related employment and earnings from municipal taxation would result in local economic development. This was supported by the data. We also hypothesized that khat sellers earn more than vegetable sellers, and that earnings for khat would be higher in the rainy season than in the dry season. We found that khat sellers do earn more overall, but that seasonal earning fluctuations followed a different pattern than expected due to the dynamics of supply and demand.

Beyond our hypotheses, we discovered challenges and opportunities of both khat and vegetable farming, revealing a dynamic complementarity that sheds light on some contours in the regional economic ecosystem. Our analysis reveals not simply why some choose to sell khat, but why some choose not to. Other studies have shown the economic impact of khat as a single commodity [6,19–21], but less attention has been paid to understanding nuances of constraints and opportunities faced by vendors in a more complex economic ecosystem. Our research fills this gap by focusing on the interplay between commodities within a local ecosystem.

Below, we contextualize the discussion of khat in its global context by examining its legal status and governance structures. This is followed by the presentation of our research and findings.

### Legal status

The legal status of khat varies widely across countries. It is legal in Ethiopia, Yemen, and Kenya, but it is banned in many Western countries, including the United States and 15 European Union member states [22]. Cathinone, the principle psychoactive substance, is amphetamine-like and has been scheduled since 1971 by the United Nations as a Schedule I drug. This means that it is determined that there is no currently accepted medical use and the potential for abuse is high. Despite this classification, the World Health Organization has “determined that the potential for abuse and dependence is low” and that existing evidence does not justify placing khat under global drug control frameworks [23].

In countries where khat is banned, legal restrictions have given rise to black markets and concerns over organized crime [22]. The United Kingdom, for example, banned khat in 2014 amid fears of addiction and social harm, though the decision was contested by researchers due to limited evidence and cultural insensitivity toward immigrant communities [11]. In a recent study from Ethiopia, khat proponents—such as local authorities, farmers, and consumers—agree on the need for legal regulation rather than prohibition [24]. In Kenya, khat bans were abandoned in 2024 following protests from farmers and sellers concerned about the potential closure of their businesses. Consequently, the President enacted national regulation and announced plans to invest in the khat industry to expand their cultivation [25].

In Madagascar, legal ambiguity around khat persists. During a 1983 conference in Antananarivo, the Interior Minister, A. Portos, acknowledged this uncertainty, stating that “*the khat question remains a subject of uncertainty for us and we need to bring some light on certain ambiguous aspects of khat*” [4]. This uncertainty persists today and can be described as a “*laissez-faire*” or “*quasilegal”* approach [10,26], as no formal legal framework currently regulates khat in Madagascar. Despite its ambiguous status, municipalities involved in khat production have established local regulations focused solely on khat tax collection. Overall, khat remains in a legal grey area in Madagascar, with its status as a licit or illicit commodity still undefined.

### Governance

Existing analyses of khat in Ethiopia suggest that khat production is a primary source of income for many communities, significantly improving livelihoods and living standards [16,21,27,28]. The economic benefits extend beyond producers to include transport workers, sellers, bar and tea sellers, and even local governments through taxation.

Governance structures in Madagascar are similar to those in Ethiopia. In both contexts, national authorities delegate regulation to local levels. One pharmacologist and public health professional suggests that any khat regulation must involve negotiation with farmers and sellers to prevent economic disruption, while ensuring broader goals such as tax revenue and employment [17]. Farmers and sellers are key stakeholders in the khat trade. Excluding them from the regulatory process can lead to resistance that undermines the implementation of local governance strategies and the collection of taxes, or *ristournes*. *Ristournes* is a form of local tax revenue that is recognized by city managers as the primary source of income for municipalities. According to Article 228, subsection 6 of Law 2014−020, the allocation of *ristournes* is regulated, distributing 60% to the Municipality and 40% to the Region. Khat-producing municipalities prioritize the collection of *ristournes*, as they represent a more substantial and reliable source of revenue than other local taxes. Effective governance also requires participatory decision-making, particularly through institutions like the *Structure Locale de Concertation* (SLC), as outlined in Article 3 of Decree No. 2015−957. These mechanisms enable municipalities and local stakeholders to collaborate on taxation policies, supporting the assertion that effective governance is rooted in local “self-regulation” [3].

Despite the absence of state or international aid in the early 2000s, Antsalaka and Joffre-ville experienced notable development driven by khat revenues. This demonstrates the crop’s local economic potential, even in the absence of external support. Nevertheless, no clear national policy exists on its governance, and regulation occurs only at the local level.

Given these dynamics, our study compares the opportunities and challenges of khat and vegetable sellers in the city of Diego-Suarez, Madagascar. While past research in Madagascar has addressed khat’s income-generating potential [13], there is a lack of comparative studies between khat and other crops. Furthermore, there are no studies of the impact of municipal taxation. This research addresses those gaps by investigating how khat trade, in comparison with vegetable trade, providing a more comprehensive analysis of how khat relatively impacts businesses income and local tax revenues.

## Methods

### Ethical clearance

The authorization to investigate the two municipalities and Diego Suarez was approved by the Head of District of Antsiranana II. The human subjects protocol was reviewed and approved by the Institutional Review Board of the University of Alabama at Birmingham (IRB 300010968).

Additional information regarding the ethical, cultural, and scientific considerations specific to inclusivity in global research is included in the Supporting Information S1 File.

The structure follows the Consolidated Criteria for Reporting Qualitative Research (COREQ) [29], which groups essential methodological elements into three domains: (i) research team and reflexivity, (ii) study design, and (iii) data analysis and reporting.

### Research team and reflexivity

The research team consisted of two principal investigators (Kotra and Gezon) and trained research assistants recruited in Diego-Suarez. The involvement of locally based team members, who share the same cultural background as the study participants, facilitated communication, trust-building, and data quality [29,30]. Kotra is a student in the Department of Management at the University of Antsiranana and conducted all interviews with city managers, as well as supervising the research assistants. The assistants were students at the same university and were familiar with local languages and market dynamics which facilitated communication and trust-building with sellers. The research assistants worked in paired teams to strengthen reliability through cross-checking observations and interpretations and to mitigate individual biases. Gezon is a professor of Anthropology at the University of Alabama at Birmingham, United States who participated in study design and analysis. She also conducted some interviews with producers and made a follow-up visit to most of the vendors in the study. This research builds on her earlier research on khat in Madagascar [31].

## Study design

### Overall approach

A mixed-methods design was used, combining qualitative interviews and ethnographic participant observation with quantitative monitoring of daily trading activities and financial records. A relatively small sample size was chosen because the study relied on a contact-intensive data collection strategy aimed at producing ethnographic thick descriptions, building trust with participants and ensuring micro-level data collection. Qualitative data were used to document practices, perceptions, and governance issues related to the khat trade, while quantitative data were used to measure earnings, prices, and seasonal variations. The two components were designed to be complementary and to allow triangulation of findings.

### Study sites and participants

Data were collected in three locations:

- Joffre-ville and Antsalaka, the two largest khat-producing municipalities where producers and municipal managers were interviewed;
- Diego-Suarez, the regional capital and economic hub where khat and vegetable sellers conduct daily trade.

Khat producers were selected through network sampling, and city managers were selected based on their institutional role and direct involvement in khat taxation and governance. Khat and vegetable sellers in Diego-Suarez were identified through purposive and network sampling. Of a total of 25 municipal neighborhoods, we identified 12 where both khat and vegetable sellers were found together and selected participants from there (See Table 1).

**Table 1:** Sample details.

Phase	Function	Women	Men	Total
**Phase I**	**City managers**	**3**	**6**	**9**
**Phase II**	**khat sellers**	**10**	**3**	**13**
	**Vegetable sellers**	**5**	**1**	**6**
**Phase III**	**khat sellers**	**11**	**2**	**13**
	**Vegetable sellers**	**7**	**0**	**7**
	**Producers**	**1**	**1**	**2**

Qualitative interviews were conducted with nine municipal officials (two mayors, two municipal treasurers, two fiscal agents, two municipal secretaries, and one deputy mayor) and two khat producers. Quantitative data collection involved 13 khat sellers and 6 vegetable sellers during the dry season (Phase II), and 13 khat sellers and 7 vegetable sellers during the rainy season (Phase III).

A relatively small sample size was chosen because the study relied on a contact-intensive data collection strategy aimed at producing thick descriptions, building trust with participants and ensuring micro-level data collection. Research assistants visited sellers at least three times per week, which supported accurate recording of daily income and pricing, and facilitated rapport-building. Saturation was achieved after repeated visits and interviews, when no new information emerged regarding pricing and earnings. The small sample size prioritized high quality, accurate data rather than high data volume with unverified and potentially unreliable data

### Participant consent and recruitment

Oral consent was obtained through an information sheet that was read and explained to the participants. It contained information about the study’s purpose, procedures, potential risks and benefits, and how participant privacy would be protected. Participants’ rights were ensured by making them aware that participation is voluntary and that subjects can withdraw at any time without penalty. Because of confidentiality concerns, participants such as city managers did not consent to audio recording; therefore, interviews were documented using written notes. Participant consent for vendors was documented by consenting to fill out the daily diaries. We attempted to maintain the same sample across the two quantitative phases; however, some participants withdrew due to external factors, particularly the Ramadan period and the aftermath of Cyclone Gamane in March 2024.

A challenge to participant recruitment was that many sellers were hesitant to participate because of khat’s legal uncertainty. Once they became aware that it was part of a university study and saw the Information Sheet for obtaining consent, they were more willing to participate.

### Data collection

This study was conducted in three phases.

In the Phase I (2022), we focused on qualitative interviews with city managers in Joffre-ville and Antsalaka. Kotra conducted face-to-face interviews, while a research assistant took notes to reduce bias [29]. Interviews were based on open-ended questions addressing khat trade governance, taxation mechanisms, municipal revenues, and impacts on local development. Municipal financial statements were also collected to assess the fiscal role of khat.

We then conducted two phases of data collection Phase II was during the dry season and Phase III during the rainy season. The rainy season lasts from December to May (6 months), characterized by hot, humid weather with risk of cyclones. The dry season lasts from June to November (6 months), with cooler temperatures and significantly less rainfall.

Data were collected from a sample of 13 khat sellers (10 women and 3 men), and 6 vegetable sellers (5 women and 1 man) during Phase II (dry season). In Phase III (rainy season), there were 13 khat sellers (11 women and 2 men), and 7 vegetable sellers (all women). (See Table 1).

Sellers documented variables including income, wholesale price, and sales volume in daily diaries. Research assistants entered the data on location using KoboToolbox and KoboCollect software on smartphones. During their visits with sellers, the team conducted participant observation and interviewed them about their perceptions of profitability, constraints, and general trade conditions.

To encourage reliability in data collection, research assistants worked in pairs. In addition to recording data in KoboCollect, they documented interactions with participants, noting emerging themes both deductively (addressing the study hypotheses) and inductively as unexpected findings arose.

## Data analysis

### Quantitative analysis

Quantitative data collected via KoboCollect were organized and analyzed using Cross Dynamic Tables (CDT) in Microsoft Excel to facilitate cross-tabulation and comparison of variables. They are particularly useful in examining the income data, since they enabled quick aggregation and comparison. SPSS 23 software was used to calculate measures of central tendency (mean and median) and variability (standard deviation and range) to provide a comprehensive overview of the economic activites of the sellers. To analyze data from Phase I, we did a financial analysis of municipal records for Joffre-ville and Antsalaka in 2019–2023.

### Qualitative analysis

Qualitative data were analyzed using narrative content analysis. Coding was performed manually through iterative readings of the transcripts and field notes, with cross-checking between researchers. The analytical approach followed a framework for generalizing case studies and incorporated Geertz’s concept of “thick description” to analyze practices at the micro-economic level [32]. Triangulation was ensured by systematically comparing interview data, observations, daily diaries, and municipal financial records.

For municipal officials and producers, the qualitative analysis was structured around Hypothesis H1, which posits that khat-related employment and municipal tax revenues contribute to local development. The coding process led to the identification of three main themes: (i) the fiscal role of khat through *ristournes* and municipal revenues, (ii) the economic role of khat in employment and livelihoods, and (iii) governance challenges related to the legal uncertainty of the khat trade.

In parallel, interviews with producers were also analyzed in relation to Hypothesis H2B, which states that khat earnings are higher during the rainy season than in the dry season. This analysis identified three domains: (i) challenges associated with khat production, (ii) risks borne by producers, and (iii) economic benefits of khat sales, with particular attention to seasonal income variations.

## Results

Our first hypothesis (H1) was confirmed, since we found through our analysis of city manager interviews that khat contributes to local development by generating employment and increasing tax revenue. *Ristournes* from the khat trade have become the main source of fiscal revenue for the two producing municipalities, showing significant growth over recent years.

We also hypothesized that khat selling would be more profitable than vegetable selling (H2A), and the results confirm this. We found, however, that while khat farmers generally earned more overall, the seasonal fluctuations in their income did not follow the expected pattern due to such factors as climate events and religious observance (H2B). Beyond our hypotheses, we found a more complex economic ecosystem than expected, revealing that, despite the higher earnings associated with khat, vegetables remain attractive to vendors who do not have the social contacts or capital to invest in khat and who seek greater market stability.

### Economic impact of the khat trade

#### Khat ristournes.

An essential dimension of the khat trade is its contribution to local tax revenue, particularly through the *ristournes* tax—a rebate mechanism that directly benefits the municipalities of Joffre-ville and Antsalaka, the principal khat-producing areas in northern Madagascar.

According to Article 181 of Law 2014−020, the resources of municipalities are categorized into:

1. Fiscal resources (e.g., Land property tax, Property tax on buildings, etc.);
2. Non-fiscal resources (e.g., *Ristournes et prélèvements,* commonly called “*ristournes*” or rebates);
3. Revenues from public or private domains (e.g., rents, exploitation rights).

Non-fiscal resources are revenues collected from economic activities within the municipality and are dedicated to both operational costs and investment in local development. In khat-producing areas, *ristournes* from khat represent the primary financial resource for municipal administrations.

#### *Ristournes* income in Antsalaka municipality.

Between 2019 and 2023, Antsalaka recorded consistently high *ristournes* revenues, with a generally positive long-term trend despite annual fluctuations. Revenues increased from 72.2 million Ariary (Ar) in 2019 to 83.4 million Ar in 2020 (+15.6%), declined slightly in 2021 and 2022, and then recovered slightly in 2023 ($1 is roughly equal to 4,500 Ar).

**Fig 1:**
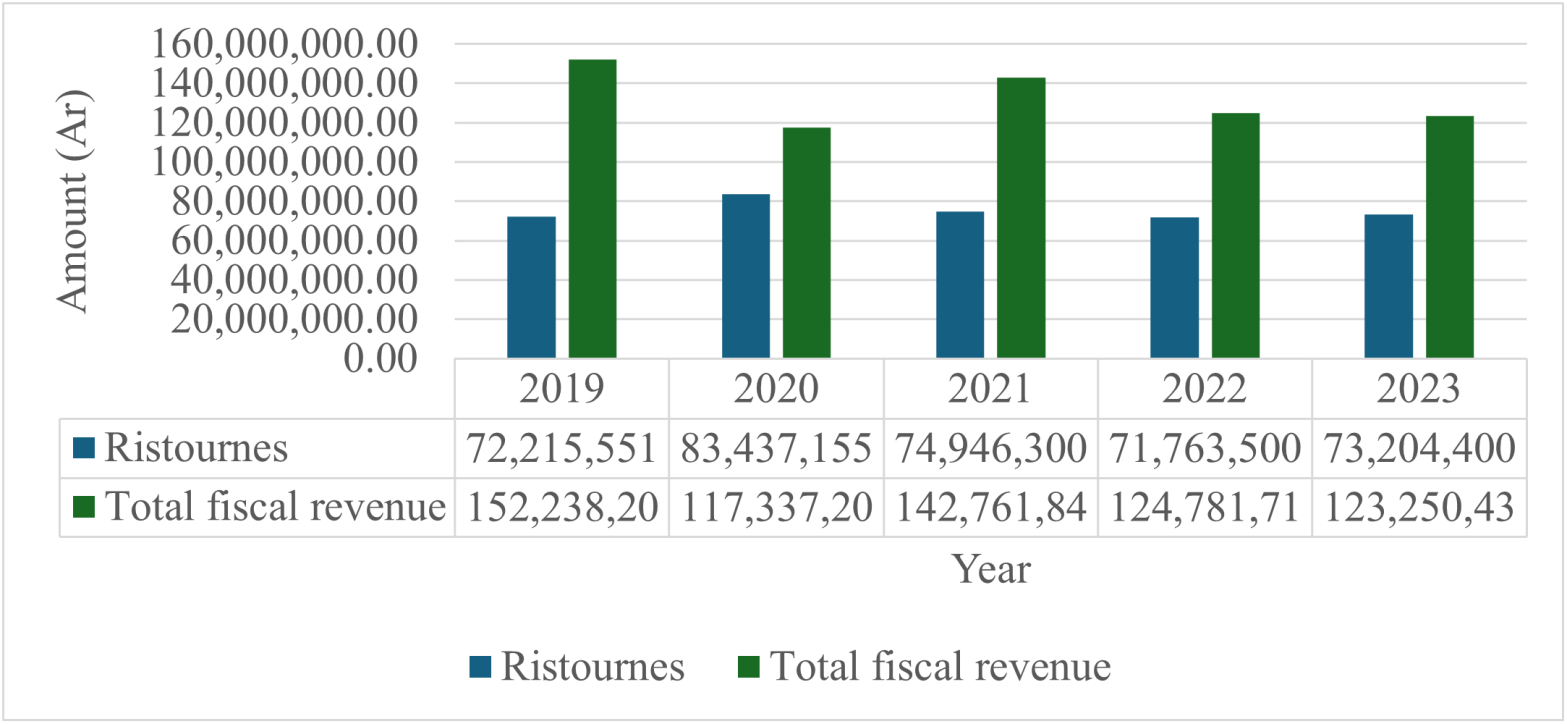
Ristournes in Antsalaka municipality.

Despite these fluctuations in absolute value, the relative contribution of *ristournes* to total fiscal revenues (including fiscal and non-fiscal revenues) remained highly significant, to constitute more than half of the local fiscal income in both 2022 and 2023 (see Fig 1).

#### *Ristournes* income in Joffre-ville municipality.

**Fig 2:**
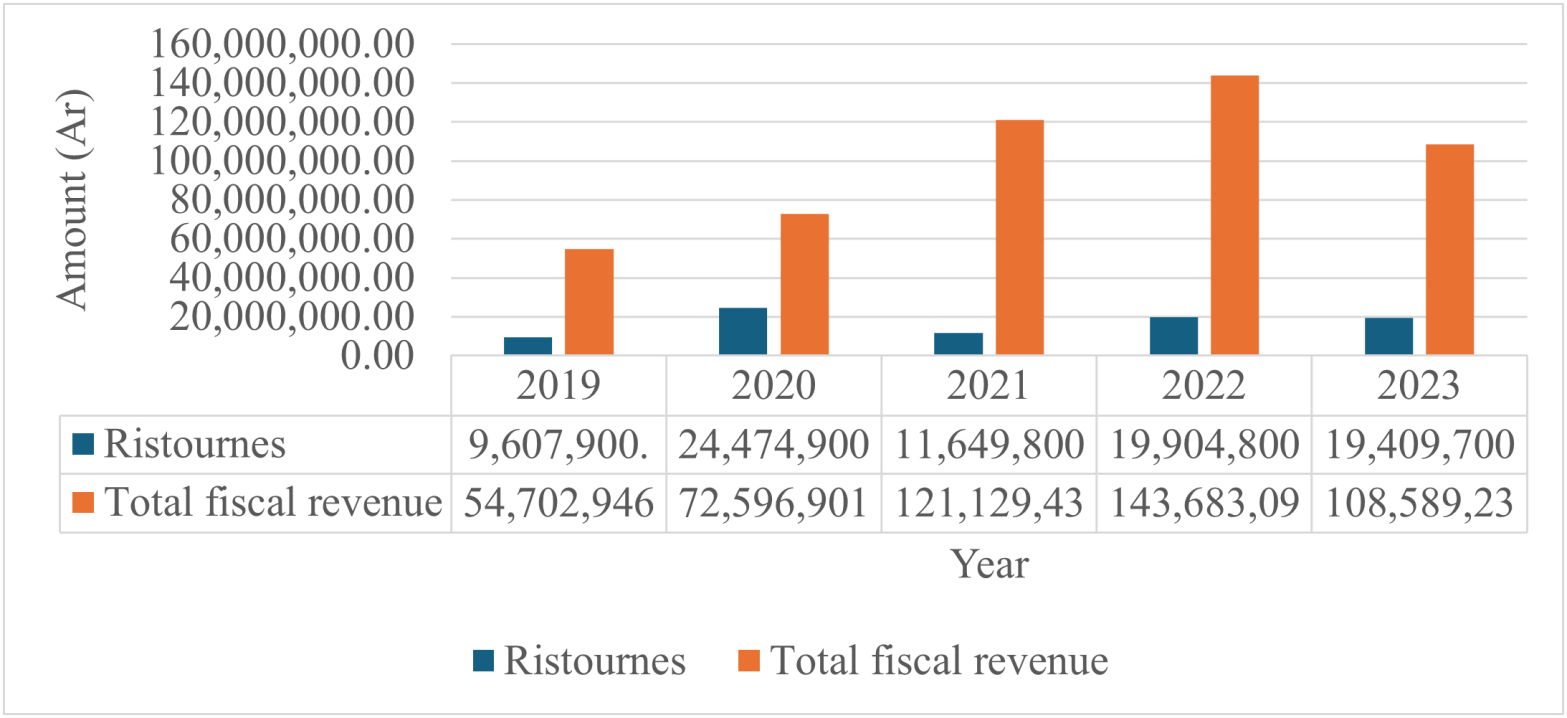
*Ristournes* in Joffre-ville municipality.

Joffre-ville exhibited a similar pattern of reliance on khat ristournes, though at lower absolute levels and with greater fluctuation, as shown in Fig 2. Ristournes revenues increased sharply from 9.6 million Ar in 2019 to 24.5 million Ar in 2020 (+154.7%), then fell in 2021, before recovering in 2022 and stabilizing in 2023. Compared to other fiscal revenues, *ristournes* account for an average of 17% of the total municipal tax income.

Although less dominant than in Antsalaka, *ristournes* from the khat trade were the largest single source of tax revenue in Joffre-ville, with other tax revenue coming from an alcohol licensing tax (*Impôt de licence sur les produits alcooliques*), event taxes (*taxes sur les fêtes ou événements*), and fees for municipal services (*prestation de services à la commune*). This progression highlights the increasing fiscal importance of khat for the municipality, even within a more diversified local economy. The growth can be attributed in part to the municipality’s decision to implement and enforce a clear policy on *ristournes* collection starting in 2019. Prior to this, there were no established rules or guidelines, and khat was not even listed as a taxable product. The rising share of *ristournes* suggests that khat’s role in local revenue generation and development financing will become more prominent in the future.

Notably, khat tax revenues remained largely unaffected by the COVID-19 pandemic in 2020. Although a slight decline was observed in 2021, this may be attributed to a temporary reduction in consumers’ purchasing power, likely due to job losses. Additionally, transportation limitations resulting from mobility restrictions may have contributed to reduced circulation of khat during that period. Despite restrictions on public gatherings, consumers continued to purchase and consume khat individually, allowing the trade—and associated tax collection—to continue uninterrupted in Diego-Suarez.

### Economic contribution of Khat *Ristournes*

The revenue generated from khat *ristournes* has enabled municipalities to invest in various infrastructure and community development projects, such as the installation of public water access points and the building of irrigation canals in Antsalaka, the construction of dedicated market shelters for khat sellers and overall village improvement initiatives in Joffre-ville. These also include support for cultural events (e.g., concerts and traditional boxing “*morengy”*) aimed at stimulating the local economy. As the Mayor of municipality 1 explained, “*the money from khat does not stay in the office; it goes back to the population through concrete projects*.” He further emphasized that accountability and feedback to taxpayers as khat sellers/producers are essential to maintain participation in the *ristourne* system: “*when sellers see where their money goes, they accept to pay. Improving the khat market was their own idea, and we realized it together*.”

The local economy in these municipalities revolves directly around khat, and market days attract a large number of vendors and customers from surrounding villages—not only for khat but also for other goods, such as electronics and satellite television subscriptions. Indirectly, the economy also benefits from khat, as these vendors pay market taxes that further support municipal finances.

### Economic outcomes of khat and vegetable trade

To fully assess khat’s role in the local economy, it is valuable to analyze it not in isolation, but rather as part of a complex and nuanced economic ecosystem. We chose vegetables as a comparator because it is another perishable commodity, because khat is perceived to be a threat to food security, and because of the concern that khat might displace the vegetable trade because of its profitability.

### Gross annual income

**Fig 3:**
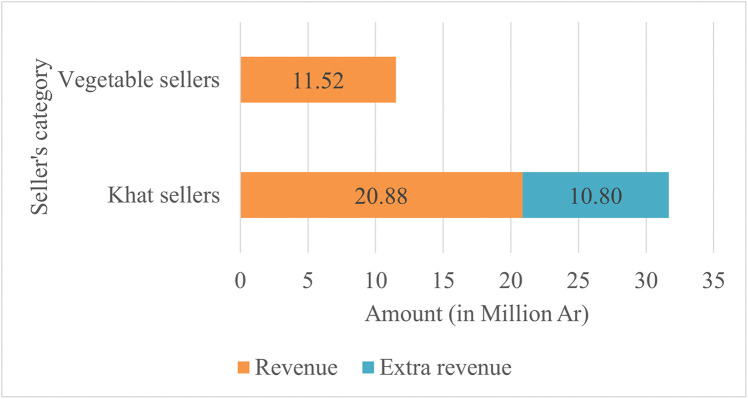
Annual income of khat and vegetable sellers.

In confirmation of our hypothesis 2A, calculations based on annualized earnings show that selling khat is significantly more profitable than selling vegetables, as seen in Fig 3 (S2 Table). Annual revenue was estimated based on data from the daily earnings diaries provided by 13 khat sellers and 6 vegetable sellers.

Selling khat is more profitable in part because vendors generate extra income by selling products that people consume alongside khat. Chewing gum and candy sales were estimated at 10 units per day at 100 Ar each. Similar estimates were applied to ginger (10 units/day at 500 Ar), cola tea (1,000 Ar per unit), cigarettes (400 Ar), and cold water (1,000 Ar) (S2 Table)These complementary sales significantly increase the total income of khat vendors as shown in Fig 3. For vegetable sellers, annualized earnings show that this trade is less profitable compared to khat. Unlike khat sellers, vegetable vendors do not generate additional income from complementary extra product sales (S3 Table).

### Net annual income: Operating costs

**Fig 4:**
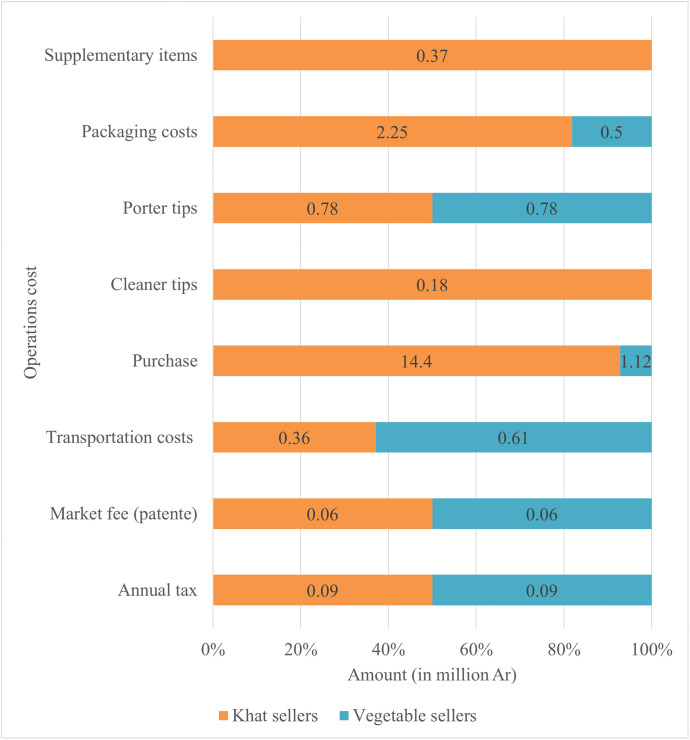
Estimated annual operating costs for khat and vegetable sellers.

To calculate net annual income, we considered key operating costs for both khat and vegetable sellers (S4 and S5 Table). These include:

- **Annual tax**: A fixed payment of 90,000 Ar per year for all vendors, paid to the regional branch of the central government before March, as mandated by the national finance law.
- **Daily market fee (“***patente***”)**: A municipal fee of 60,000 Ar that grants all vendors legal rights to operate at designated sales points.
- **Transportation costs**:
  For khat sellers, transport is often included in the wholesale price. However, vendors may pay an additional 2,000 Ar/day for local delivery from the wholesale point to their stalls. To calculate annual expenses, we estimate that sellers pay for transportation 180 days per year.
  For vegetable sellers, transportation is more expensive. Wholesalers sourcing from the region surrounding the capital city of Antananarivo, were the majority of vegetables are grown, spend about 36,000 Ar per supply trip. Retailers operating within Diego-Suarez pay around 2,000 Ar daily using local taxis. Supply frequency depends on sales volume—daily for retail sellers, and weekly for wholesalers.
- **Tips for porters**: When help is needed for moving goods, all vendors pay a median of 6,500 Ar per trip. Based on our data from daily diaries and discussions with sellers, we estimate that they use porters 120 days per year.
- **Packaging expenses**:
  Khat sellers spend a median of 12,500 Ar per purchase for specialized packaging (boxes or plastic bags), sometimes charging customers for bags.
  Vegetable sellers spend less—about 2,750 Ar per purchase—and typically include packaging in the sale price.
- **Purchase of supplementary items for sale (for khat sellers)**: These include cold water, chewing gum, cola tea, and similar products consumed with khat.
- **Tips for cleaners (khat sellers only)**: In Diego-Suarez, known for its municipal cleanliness, khat sellers may pay street cleaners (often informal workers or friends) around 1,000 Ar/day/person. Based on daily diaries, we calculate they incur this cost only 180 days per year. While some clean their own areas to save costs, municipal agents inspect the cleanliness of sales points. Vegetable sellers do not usually incur this cost.
- **Product purchase costs**: The largest proportion of expenses is attributed to the purchase of the products, as shown in Fig 4.
  For khat, the median wholesale purchase price per day per vendor fluctuates seasonally, at 30,000 Ar in the rainy season and 50,000 Ar in the dry season.
  For vegetables, median wholesale purchase prices vary, at 5,000 Ar in the rainy season and 1,200 Ar in the dry season. Purchases are usually made weekly or daily, depending on sales volume and stock.

**Fig 5:**
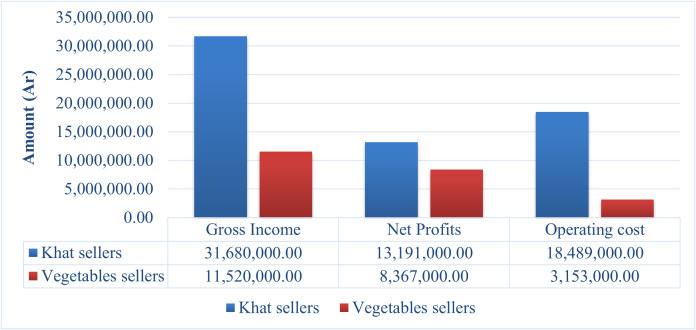
Comparison between khat and vegetable sellers.

These findings reveal that some choose to sell vegetables instead of khat because of the lower cost of the capital investment.

### Earning variability among sellers

**Fig 6:**
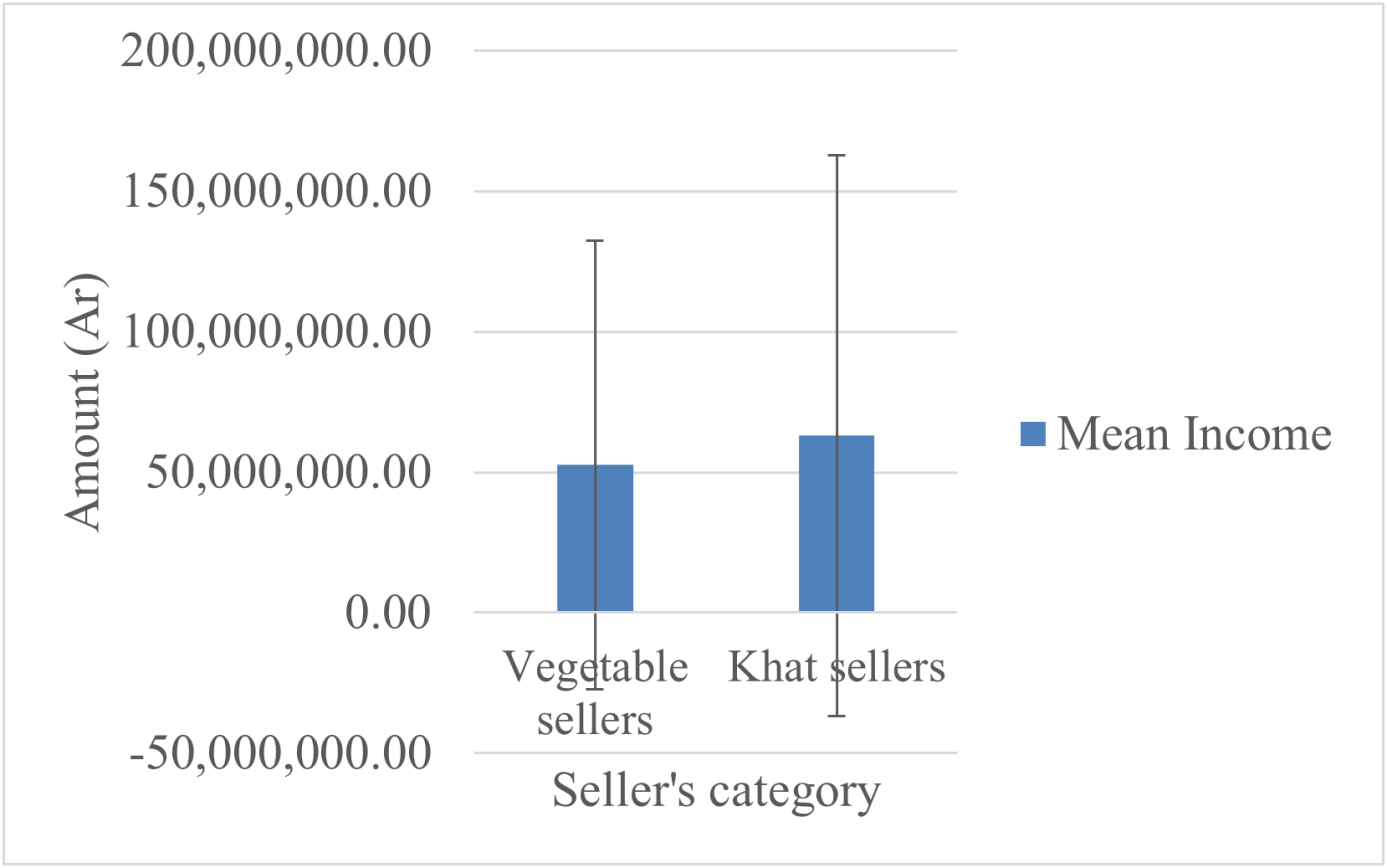
Annual Income Variability.

There is income variability among khat and vegetable sellers. Overall, vegetable sellers earned on average 52.6 million Ar per year, while khat sellers earned 63.1 million Ar. However, the large standard deviations observed in Fig 6 for both categories (observed in the vertical lines on the‌‌ graph) indicate substantial heterogeneity among vendors, confirming that income levels vary widely within each group (See details on S6 Table). The range of profitability is particularly large for khat sellers, meaning that some earn vastly more than others. Part of this is because some in each category are retailers only (with smaller scale and local markets), while other retailers may also be larger wholesale sellers, who likely benefit from economies of scale and have more power to set their price.

### Price fluctuations

Our hypothesis was confirmed that overall earnings from khat were higher in the rainy season than in the dry season (H2B). This is due in large part due to supply and demand: There are larger quantities of khat during the rainy season because of increased plant growth and because farmers without irrigation can grow it. The increased quantity of khat results in lower prices for consumers and increased volume of sales for vendors. This contributes to the widely held perception that khat prices are seasonal—higher in the dry season when production is lower and availability is limited [33]. We found, however, that despite lower prices in the rainy season, vendors earned more overall because of the higher volume of sales (see Table 2).

**Table 2:** Fluctuation of median wholesale purchase prices and income in Ariary.

Category	Item	Rain season	Dry season
**Khat**	**Purchase price**	30,000.00	50,000.00
	**Gross income**	60,000.00	56,000.00
	**Sales volume**	2 bunches/day/seller	1.12 bunches/day/seller
**Vegetables**	**Purchase price**	5,000.00	1,200.00
	**Gross income**	40,000.00	24,000.00
	**Sales volume**	8 kg/day/seller	20 kg/day/seller

### Khat’s price fluctuations

**Fig 7:**
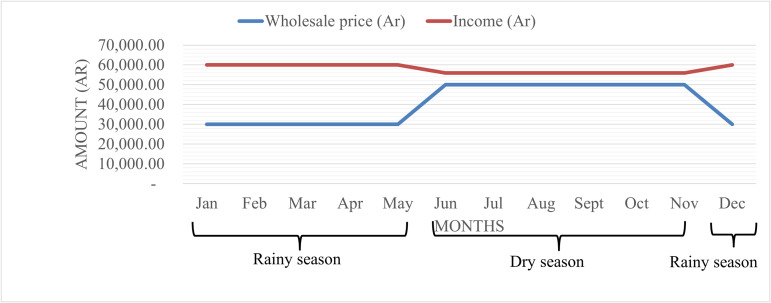
Fluctuation of wholesale price of khat during a year.

The price sellers pay for khat fluctuates across seasons (see Fig 7). The average wholesale purchase price per unit is lower in the rainy season at 30,000 Ar per bunch, while it increases to 50,000 Ar per bunch in the dry season. Although sellers and consumers alike pay less for khat in the rainy season, the daily median income per vendor is higher 60,000 Ar in the rainy season (60,000 Ar), compared to the dry season (56,000 Ar) because more people can afford to purchase khat. With khat, lower prices mean higher sales.

The reasons for fluctuations were more nuanced than simply supply and demand related to seasonal weather patterns, however. We also found seasonal price fluctuations due to religious observance. The Muslim period of fasting, Ramadan, occurred during the dry season in the year of our study, resulting in an unexpected increase of prices. Producer 1 explained, “*sometimes, I decided not to sell during Ramadan because we have fewer clients, since most of our customers are Muslim.*” This statement helps to explain our quantitative results showing that, during the dry season, gross income decrease for khat sellers. Several vendors anticipated a reduction in demand due to fasting and therefore temporarily stopped selling khat. However, unexpectedly, on weekends prices went up. As another seller noted, “*during Ramadan, when people come on weekends, I increase my price to fill the gap during the week*.” These quotes illustrate how religious calendars interact with market behavior, shaping both selling strategies and price dynamics.

### Vegetable’s price fluctuations

**Fig 8:**
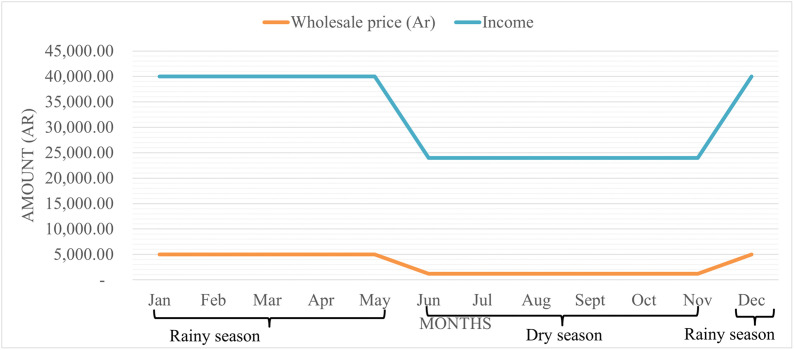
Fluctuation of wholesale price of vegetables during a year.

Vegetable sellers earned more in the rainy season during our study period, despite paying more for their product and selling fewer kilograms per day (see Table 2). In the dry season (June to November), the median wholesale purchase price of a unit of vegetables was 1,200 Ar, while the gross income was 24,000 Ar. per day (see Fig 8). In contrast, during the rainy season (December to May), the purchase price rose to 5,000 Ar and the gross income increased to 40,000 Ar. While transportation costs are always higher for vegetables during the rainy season, this was even more so during our study period, since the Cyclone Gamane washed out a bridge over a major river on the only main road between Antananarivo and Diego Suarez. This resulted in delivery delays and higher costs, as transporters had to hire boats to move goods (as a transportation substitute in the absence of a bridge).

In contrast with khat, where higher dry season prices resulted in lower gross earnings, higher vegetable prices did not result in decreased gross earnings because they offset their expenses by increasing the sale price (see Table 2). This higher willingness to pay higher costs for vegetables suggests that people were willing to pay more for a food staple than for khat, which may be viewed as recreational. Additional research would be needed to investigate this relationship.

## Discussion and recommendations

These findings confirm previous research that identifies khat production and trade as beneficial for local economies by strengthening household resilience and generating meaningful contributions to local development [16,21,27,28]. This confirms our hypothesis that khat boosts local economic development in northern Madagascar by generating employment and municipal taxes that support infrastructure and local development (H1). Further, khat selling proved to be more profitable than vegetable selling (H2A), with higher overall earnings despite seasonal price fluctuations (H2B). We found that vendors with family ties to khat farmers earn considerably more than others. In addition to our hypotheses, we found that khat plays a complementary role in the local economic ecosystem and that there are economic reasons why people continue to sell vegetables despite khat’s greater profitability.

### Khat and economic development

Many studies have shown that khat helps reduce poverty [34,35], highlighting khat’s critical economic role, especially in low-income contexts [36]. In Kenya, researchers [5,36] highlight its role in sustaining rural economies. Similar findings are reported in Ethiopia [6,7,17,27,35,37]. Yemen also recognizes the khat sector as a major economic force like the khat sector produces 6% of national GDP in 2008 [3] and 10% of the national GDP in 2010 [10].

Several scholars [28,38,39] found that households engaged in khat cultivation enjoy higher living standards than those who do not. Others like [17] document that khat contributes to enhanced quality of life among producers, contributing not only to individual income but also to broader local economic growth. Some [13] argue that the khat economy has led to improved living conditions for those involved in its production and trade. Khat also significantly boosts income for khat sellers, contributing to employment and income in the countries where it is grown and sold [6,21,34]. Our findings in Madagascar confirm the benefits to both producers and sellers who tended to have relatively significant incomes and purchasing power and, in some cases, improved access to services and infrastructure. The data validate our initial assumption that khat yields relatively high profits (see Fig 5), positioning it as an attractive and strategic commodity in local markets for some vendors.

In addition to individual and household earnings, the khat economy also contributes significantly to municipal development through tax revenue. Our analysis shows that “*ristournes*” (rebates) from the khat trade provide a critical source of funding for local governments (see Figs 1 and 2). Furthermore, sellers contribute to the national tax system through formal taxation mechanisms (see Fig 4), reinforcing the khat sector’s role in public finance and institutional development.

### Relative advantages of khat and vegetable selling

Our research reveals that while earnings from selling khat are 58% higher than from selling vegetables, selling vegetables has distinct advantages for some. As such, both commodities hold an important place in the local economic ecosystem.

Vegetable vending has distinct financial disadvantages. First, there is a lower profit margin and a higher risk of financial loss due both to the difficulty of storing the product without refrigeration and the competition between vendors. Vegetable sellers are concentrated in crowded markets, leading to reduced pricing flexibility and fewer customers per vendor. There are also logistical constraints: most vegetables come from Antananarivo, approximately 754 kilometers away. This long-distance supply chain is vulnerable to road conditions and weather disruptions. This was illustrated during our study period when a cyclone destroyed a key bridge, sharply reducing the supply of vegetables and inflating prices. Higher retail prices helped the vendors recuperate their investments, however, and gave them higher average income than in the dry season.

Nevertheless, we learned that vegetables remain attractive to many vendors for several reasons. First, the barrier to entry for selling vegetables is low, requiring small capital investments and the ability to purchase from wholesalers within the city. Furthermore, the willingness to pay for vegetables despite higher costs suggests relative market stability. Despite the higher overall earnings from selling khat, vegetable selling appears to be a steadier source of income.

Selling khat has distinct and obvious advantages, the most important being the significantly higher profit margin. Khat is even more perishable than vegetables, as it loses its potency after one day. Nevertheless, the high demand for khat and the geographic dispersion of vendors throughout the city make it easier for them to sell their product each day and avoid waste. Logistically, khat is sourced locally, purchased from farms located just 31.6 to 35.5 kilometers away (Diego-Suarez to Joffre-ville and Antsalaka), which reduces transportation costs and makes it easier to obtain advantageous wholesale relationships due to direct relations between vendors and producers.

However, these benefits are coupled with higher operating costs, greater capital requirements, the need to travel out of the city to purchase wholesale at the best price, and social barriers to entry. What is an advantage for khat selling can be a disadvantage for some: New entrants often rely on connections to producers or wholesalers to negotiate better purchase prices. Conversely, vegetable sellers face lower social and financial barriers to entry because of the lower wholesale commitment and the more transactional relationships with wholesalers.

In sum, while khat selling offers clear economic advantages, these are not universally accessible. This explains why vegetables remain a vital and sustainable component of the local economy: they serve different functions and fill distinct niches within the marketplace ecosystem.

## Governance and recommendations

The governance and legality of khat remain a complex issue, shaped by competing economic, social, and health considerations. A pharmacologist has noted that the literature frequently either exaggerates the dangers of khat due to its unfamiliarity or minimizes its effects because of its cultural importance [40]. This tension creates challenges for both researchers and policymakers in assessing khat’s overall value. As a psychoactive substance, khat presents health, social, and environmental challenges that some argue justify its prohibition and scheduling as a drug of abuse [36].

On the other hand, the khat trade contributes to achieving Sustainable Development Goal (SDG) 1, which aims to eradicate poverty in all its forms, by enhancing household income, supporting municipal revenues, and creating opportunities for small-scale entrepreneurship. We join others in arguing that banning or neglecting the khat industry could exacerbate poverty for many individuals who depend on it for their livelihoods, particularly in low-income countries like in Kenya, Yemen, in Ethiopia and Madagascar [15,36,39].

Because of the significance of khat’s economic impact, khat prohibition would likely cause socioeconomic harm, as seen in Somaliland [14]. Instead, we argue that khat should be treated similarly to other legal psychoactive crops like coffee, tobacco, and tea, through regulated legality that addresses public health and harm reduction without undermining livelihoods.

Thus, local authorities emphasized that they must constantly seek a balance between the economic benefits and the social consequences of khat consumption, The mayor of municipality 2 stated that “*Governing development from khat trade and its taxation is not easy, we have to think about the balance between the economic impact and the social impact of khat*,” Additionally, another municipal official added that “*working together on local development does not only create economic impact, it also builds trust between traders and local authorities*.” While acknowledging the central economic role of this activity, he nevertheless stressed the need for regulation, adding that “*banning khat is not an option for me, but we need to think about some regulation to limit young people’s access*.” These quotes illustrate that compliance with *ristourne* payments is not based solely on coercion, but on a reciprocal relationship grounded in visible accountability enhancing good governance.

## Addressing concerns

### Health and social concerns

Health and social concerns should be addressed by examining their root causes instead of their superficial manifestation in khat production and consumption. Social issues, for example, include concerns that people, and youth in particular, are choosing to chew khat instead of taking jobs. These concerns need to be examined within the context of chronic unemployment and lack of recreation opportunities [41], with a focus on job creation. There are also concerns that consumers spend too much on khat instead of household essentials, including food and children’s education [3,6,42,43]. These valid concerns need to be examined in the context of addictive and recreational spending behavior in general, considering similar effects of coffee, tobacco and alcohol consumption. Health concerns can be addressed through harm reduction strategies, as they are with tobacco in much of the world [44], focusing on education and social support, and by increasing the capacity of healthcare services. Legal frameworks could draw on existing models for regulating substances like alcohol and cigarettes, including taxation, pricing strategies, and educational campaigns to curb excessive consumption, especially among youth.

### Economic concerns: Food security and livelihood diversification

Some scholars argue that khat improves food security by increasing purchasing power [16,33,36,39]. Others disagree, warning that khat undermines crop diversity and increases food insecurity by encouraging monoculture [28,39]. Indeed, some vegetable sellers complained about sourcing vegetables from the capital due to regional shortages, which has raised prices and reduced local access to nutritious food. To protect food security, scholars have recommended regulating khat’s expansion while promoting the viability of crop diversification and alternative livelihoods [27]. We agree with this approach, which aims to mitigate the trade-offs between economic gain and long-term food security.

There is indeed a risk of over-reliance on the khat industry because of the potential impact on food security and vulnerability to other market and supply fluctuations. One recommendation for improving the viability of vegetable commodities and increase food security is to invest in local productive capacity rather than rely on purchasing from Antananarivo. Cooperative marketing could embrace diverse viewpoints to best identify obstacles and opportunities for sustainable outcomes [45]. Another recommendation is to for the city to increase the viability of vegetable selling by developing spacious market spaces throughout the city. Another approach is to encourage khat farmers to diversify into other agricultural or non-agricultural activities. This approach would support a more sustainable, long-term strategy and reduce dependence on a single product [35].

Because of its current and potential economic impact, we recommend generating local fiscal policies that strengthen tax recovery and coordinate between regional and municipal tax agents [1,21]. Better enforcement could also combat tax evasion and enhance local development.

Increasing taxes on khat could be an effective strategy to reduce consumption by weakening its market accessibility. Raising the tax on khat would serve two purposes. First, higher taxes would raise the price of khat, potentially reducing demand, especially among youth and lower-income populations. Second, the increased tax revenue would benefit the municipality by strengthening its financial autonomy. This would reduce dependence on external donors and state funding, allowing the municipality to have greater control over investments and allocate resources based on the real needs of its citizens.

### Environmental concerns

Finally, khat production raises concerns about deforestation and the potential for destruction of watersheds, threatening water scarcity in the city of Diego-Suarez. Local authorities attribute water shortages to excessive khat irrigation, while producers point to declining rainfall. This tension calls for evidence-based, proactive watershed management policies and practices [1,39].

In sum, we argue that khat should be treated similarly to other legal psychoactive substances such as tobacco or coffee, with public health and agricultural interventions tailored accordingly. Local municipalities could consider: (i) strengthening agreements on urban cleanliness; (ii) increasing the khat tax rate and tax recuperation efforts to optimize municipal revenue; and (iii) regulations designed to reduce youth access. These suggestions mirror those who advocate for public health strategies to mitigate overuse of khat in Ethiopia [27]. Similarly, others [39] recommend investment in agricultural support or substitution systems, including technology transfers and improved irrigation infrastructure, to strengthen rural economies while reducing dependence on khat.

Banning khat in regions where it is culturally embedded is unlikely [46]. Rather, regulation, formalization, and taxation [47], could help legitimize the industry, enhance government revenue, and reduce informal market risks.

## Conclusion and limitations

This study offers a mixed-methods, analysis of the khat trade in northern Madagascar, contributing nuanced understanding to a debate often dominated by macro-level assessments focusing on khat as a single, isolated commodity. While previous literature has broadly outlined khat’s economic benefits, our micro-level approach sheds light on the day-to-day realities of sellers, particularly regarding price fluctuations, comparative market dynamics, and barriers to entry.

Some limitations raise questions about the generalizability of our study. The limitation of a small sample size was mitigated through in-depth qualitative research, which allowed for triangulation of the findings through informal interviews and observations on the ground. Another limitation was the complexity of the phenomena studied, which required regular team meetings aimed at increasing the validity and reliability of data collection. Finally, the data were collected during a single year, making it difficult to assess the impact of extreme disruptive events before and during data collection, such as the cyclone Gamane.

Overall, despite the limitations, we feel confident in asserting that our findings confirm that khat plays a central and complementary role in the local economy, offering sellers meaningful opportunities for income generation and financial autonomy. For future research, we envision doing a longitudinal study of price and profit variability, considering their impact on quality-of-life measures. We also plan a study of the role and impact on women of the khat and vegetable economy.

## Supporting information

S1 File PLOS inclusivity in global research questionnaire. (PDF)

S1 Table Annual income statement of khat sellers. (DOCX)

S2 Table Annual income statement of vegetables sellers. (DOCX)

S3 Table Estimated annual operating costs for khat sellers. (DOCX)

S4 Table Estimated annual operating costs for vegetable sellers. (DOCX)

S5 Table Annual income variability. (XLSX)
