# Supplementary material for: An assessment of the khat and vegetable trade in the local economic ecosystem: The case of northern Madagascar
Source: PLoS One. 2026 Jun 11;21(6):e0331722. doi: 10.1371/journal.pone.0331722 (PMC13257991; doi:10.1371/journal.pone.0331722)
Supplement: S1 Table — (DOCX) [file pone.0331722.s002.docx]

Table. Annual income statement of khat sellers

| **Income** | **Amount (Million Ar)** | **Formula** |
| --- | --- | --- |
| **Revenue** | 20.88 | Dry season: 56,000 Ar × 30 days × 6 months + Rainy season: 60,000 Ar × 30 days × 6 months |
| **Extra revenue** | | |
| Chewing-gum and candy | 0.36 | 10 units/day × 100 Ar × 360 days. Estimated at 10 pieces/day sold at 100 Ar each (Maximum price) |
| Ginger | 1.80 | 10 units/day × 500 Ar × 360 days. Estimated at 10 units/day sold at 500 Ar each (Maximum price) |
| Cola tea | 3.60 | 10 units/day × 1,000 Ar × 360 days. Estimated at 10 units/day sold at 1,000 Ar each (Maximum price) |
| Cigarettes | 1.44 | 10 units/day × 400 Ar × 360 days. Estimated at 10 units/day sold at 400 Ar each (Maximum price) |
| Cold water | 3.60 | 10 units/day × 1,000 Ar × 360 days. Estimated at 10 units/day sold at 1,000 Ar each Maximum price) |
| **TOTAL** | **31.68** |  |
| **Profits** | **13.19** |  |
| **Monthly profits** | **1.10** |  |
