# Supplementary material for: An assessment of the khat and vegetable trade in the local economic ecosystem: The case of northern Madagascar
Source: PLoS One. 2026 Jun 11;21(6):e0331722. doi: 10.1371/journal.pone.0331722 (PMC13257991; doi:10.1371/journal.pone.0331722)
Supplement: S2 Table — (DOCX) [file pone.0331722.s003.docx]

Table. Annual income statement for vegetables sellers

| **Income** | **Amount (Million Ar)** | **Formula** |
| --- | --- | --- |
| Revenue | 11.52 | Dry season: 24,000 Ar × 30 days × 6 months + Rainy season: 40,000 Ar × 30 days × 6 months. |
| **TOTAL** | 11.52 |  |
| **Net Profit** | **8.37** |  |
| **Monthly profit** | 0.70 |  |
