# Supplementary material for: An assessment of the khat and vegetable trade in the local economic ecosystem: The case of northern Madagascar
Source: PLoS One. 2026 Jun 11;21(6):e0331722. doi: 10.1371/journal.pone.0331722 (PMC13257991; doi:10.1371/journal.pone.0331722)
Supplement: S3 Table — (DOCX) [file pone.0331722.s004.docx]

Table. Estimated annual operating costs for khat sellers

| **Expense** | **Amount**  **(Million Ar)** | **Formula** |
| --- | --- | --- |
| Annual tax | 0.09 | Fixed, per national finance law |
| Market fee (*patente*) | 0.06 | Fixed, set by municipality |
| Transportation costs | 0.36 | 2,000 Ar × 180 days (estimated for half the year due to non-daily purchases). |
| Khat purchase | 14.40 | Dry season: 50,000 Ar × 30 days × 6 months + Rainy season: 30,000 Ar × 30 days × 6 months |
| Cleaner tips | 0.18 | 1,000 Ar × 180 days (Cleaners not hired daily; many sellers clean their own points). |
| Porter tips | 0.78 | 6,500 Ar × 120 days (Only some sellers use porters occasionally). |
| Packaging costs | 2.25 | 12,500 Ar × 180 days (Packaging needed occasionally; estimate assumes 50% of working days) |
| Supplementary items | 0.37 | 2,050 Ar × 180 days (Includes personal stock for resale (e.g., cola tea, gum); not purchased every day) |
| **TOTAL** | **18.49** |  |
