# Supplementary material for: An assessment of the khat and vegetable trade in the local economic ecosystem: The case of northern Madagascar
Source: PLoS One. 2026 Jun 11;21(6):e0331722. doi: 10.1371/journal.pone.0331722 (PMC13257991; doi:10.1371/journal.pone.0331722)
Supplement: S4 Table — (DOCX) [file pone.0331722.s005.docx]

Table. Estimated annual operating costs for vegetables sellers

| **Expense** | **Montant (Million Ar)** | **Formula** |
| --- | --- | --- |
| Annual tax | 0.09 | Fixed, per national finance law |
| Market fee (*patente*) | 0.06 | Fixed, set by municipality |
| Transportation costs | 0.61 | 36,000 Ar × 17 weeks (Estimated for one-third of the year. Most vegetable sellers purchase once per week). |
| Vegetable purchase | 1.12 | Rainy season: 5,000 Ar × 30 days × 6 months + Dry season: 10,000 Ar × 30 days × 6 months. |
| Porter tips | 0.78 | 6,500 Ar × 120 days (Porter services are not used daily. Estimated for one-third of the year). |
| Packaging costs | 0.50 | 2,750 Ar × 180 days (Packaging costs are not incurred daily. Estimated for half the year). |
| **TOTAL** | **3.15** |  |
