# Supplementary material for: An assessment of the khat and vegetable trade in the local economic ecosystem: The case of northern Madagascar
Source: PLoS One. 2026 Jun 11;21(6):e0331722. doi: 10.1371/journal.pone.0331722 (PMC13257991; doi:10.1371/journal.pone.0331722)
Supplement: Attachment — Submitted filename: PLOS ONE_paper revision_22_10_2025.pdf [file pone.0331722.s007.pdf]

Peer Review PLOS ONE Khat and the economic ecosystem in northern Madagascar

The Introduction presents a well-researched overview of khat's botanical identity, cultural relevance, and economic role, especially in Madagascar. It effectively transitions from global context to local focus and includes relevant legal and governance aspects. However, the section would benefit from clearer structure, more concise language, and a stronger articulation of the research gap. Refining paragraph transitions and avoiding redundancy would improve readability, while briefly referencing the methodological approach would help orient the reader. The introduction would benefit from a more explicit statement of the research gap and a brief mention of the methodological approach to better orient the reader. Overall, while the introduction is rich in content and well-cited, it requires editorial refinement to enhance clarity, coherence, and scholarly impact.

The Methods section presents a thorough and context-aware account of the study's design, including ethical approvals, sampling strategies, data collection, and analysis. It reflects a careful approach to fieldwork in a legally and socially sensitive environment, combining qualitative and quantitative methods appropriately. The authors acknowledge recruitment challenges and describe efforts to build trust with participants, which strengthens the credibility of the data. However, the section would benefit from clearer organization, with labeled subsections to improve readability, and more consistent terminology. The use of COREQ is mentioned but not explained, and the rationale for sample size could be supported by a clearer discussion of saturation criteria. Overall, the section is methodologically sound but could be refined for clarity and completeness.

The Results section presents a detailed and well-organized analysis of khat's economic role in northern Madagascar, particularly its contribution to municipal tax revenue and vendor income. The findings are clearly linked to the study's hypothesis and supported by rich fiscal data and seasonal comparisons. However, the section could be improved by simplifying repetitive narrative elements, clarifying the integration and interpretation of figures and tables, and refining the structure with clearer subheadings. The contrast between khat and vegetable selling is compelling, especially in terms of profitability and barriers to entry, but would benefit from deeper qualitative insights and clearer explanation of cost structures. Overall, the section is strong in content but would gain from editorial refinement and more effective data presentation.

The Discussion and Recommendations section offers a well-rounded synthesis of the study's findings, effectively linking them to existing literature and broader development goals. It confirms the central role of khat in local economic development and explores its complementary relationship with vegetable selling. The authors address health, social, environmental, and food security concerns with nuance, proposing regulation over prohibition and aligning their recommendations with Sustainable Development Goal 1. While the section is grounded in empirical evidence and avoids simplistic conclusions, it would benefit from clearer structure and deeper engagement with potential risks, such as over-reliance on khat, legal ambiguity, and food insecurity. Expanding on how increased tax revenues could support public services and offering more concrete examples would strengthen the policy relevance of the recommendations.

The Conclusion provides a concise and coherent summary of the study's main contributions, emphasizing the value of a micro-level, ethnographic approach to understanding the khat trade in northern Madagascar. It reinforces the finding that khat plays a complementary role in the local economy and highlights barriers to entry for different types of vendors. Limitations such as the small sample size and the one-year data collection period are acknowledged with transparency. While the section is well-aligned with the rest of the manuscript, it could be strengthened by suggesting directions for future research, such as longitudinal studies or comparative analyses, to deepen understanding and enhance generalizability.

Minor grammatical issues should be corrected throughout the manuscript. For instance, in the Introduction, “1 men” should be revised to “1 man”; “Sellers’type” to “Sellers’ type”; and “because of his legal uncertainty” likely refers to “because of its legal uncertainty.” These corrections should be applied consistently across all sections. A thorough grammar and style revision is recommended to ensure smoother flow and greater clarity.
