# Supplementary material for: An assessment of the khat and vegetable trade in the local economic ecosystem: The case of northern Madagascar
Source: PLoS One. 2026 Jun 11;21(6):e0331722. doi: 10.1371/journal.pone.0331722 (PMC13257991; doi:10.1371/journal.pone.0331722)
Supplement: Attachment — Submitted filename: Response to reviewers_khat paper_Kotra and Gezon.pdf [file pone.0331722.s009.pdf]

KOTRA Mahandrisoa Candicia,
candiciakotra@gmail.com
+393513699559/+261328267489
University of Antsiranana, Madagascar

December 12th , 2025

Subject: Submission of Revised Manuscript [PONE-D-25-38409] - [EMID:3f1a047343d21f64] – Response to Reviewers for "Khat and the economic ecosystem in northern Madagascar".

Dear Editorial Committee,

Thank you for your response dated November 3rd 2025 regarding our manuscript, **Khat and the economic ecosystem in northern Madagascar - PONE-D-25-38409**, which you have conditionally accepted pending minor revisions. We appreciate the time and effort the reviewers dedicated to evaluating our work; their insightful comments have been invaluable in strengthening the manuscript.

We have carefully considered all comments and suggestions provided by the reviewers. Our manuscript has been thoroughly revised according to their feedback. Our primary changes include :

- Clarifying the methodological approach in the Abstract and Introduction while avoiding redundancy.
- Improving data presentation in figures 1-6 and tables 1-2 with clear description.
- Expanding the discussion/explanation on policy recommendations according to the reviewer's comments.
- Following Plos One guidelines for Editorial format about captions for the Supporting Information files at the end of the manuscript and dataset availabilty.

For your convenience, we have prepared a point-by-point response to every comment raised by Reviewers #1, #2, and #3. This detailed response is provided in the appended document, where we indicate the specific location (section) of the changes in the revised manuscript.

We believe that these revisions have substantially improved the clarity, coherence, and scholarly impact of our article, making it suitable for publication in the PLOS ONE.

We look forward to hearing from you regarding the suitability of the revised manuscript.

Sincerely,

KOTRA Mahandrisoa Candicia

Reviewer	Comments	Location in Article and Comments
**Reviewer #1**	Authors should provide more details on the mixed-methods approach beyond just qualitative and quantitative methods in the abstract.	Details were added on Method section
Reviewer #1	Clarify the different scientific names for *Catha edulis* (Forssk, Forsk, Forskal) used in the introduction for consistency.	Deleted confusing and unnecessary wording about the scientific name and the plant's origins. In Introduction section
**Reviewer #1**	Elaborate on the dynamic complementarity between khat and vegetable farming to provide a clearer understanding of their intertwined roles in the economic ecosystem.	This dynamic complementarity is first presented in the "Study Purpose and Hypotheses" section and elaborated in the discussion in the section "Relative advantages of khat and vegetable selling"
**Reviewer #1**	Provide more context or a brief explanation for the use of cross-dynamic tables in data analysis, as it might not be a universally familiar term.	Explanation of cross dynamic tables is given right under the "Data Analysis" heading.
**Reviewer #1**	When discussing the exponential growth trend of khat ristournes collection, provide a brief explanation of what ristournes are for readers unfamiliar with the term.	A description of ristournes was added in the section "Governance" heading
**Reviewer #1**	Ensure consistency in the description of ristournes as both "rebates and tax levies" or just "tax levies" throughout the text for clarity.	We identified them as ristournes or rebates consistently.
**Reviewer #1**	Strengthen the discussion on how the study's findings specifically confirm previous research, rather than just stating it generally.	Citations are added under Discussion and recommendations.
**Reviewer #1**	All the figures must be written with clear labeling and captions instead of giving a title in the figure itself.	This was done.
**Reviewer #1**	The authors should offer more detailed examples or mechanisms that demonstrate how local fiscal policies can enhance tax recovery and coordination.	See section "Addressing concerns/ Economic concerns: Food security and livelihood diversification"

**Reviewer #1**	Elaborate on the proposed regulated legality for khat, outlining what specific regulations may include addressing public health and harm reduction.	See section "Addressing concerns/ Health and social concerns"
**Reviewer #1**	Refer to the paper "Collaborative Marketing Strategies in Agriculture for Global Reach and Local Impact." Emerging Trends in Food and Agribusiness Marketing, edited by Satish Chandra Pant, et al., IGI Global Scientific Publishing, 2025, pp. 219-252.	This has been added as reference 45.
**Reviewer #2**	The Introduction section would benefit from clearer structure, more concise language, and a stronger articulation of the research gap.	We streamlined details about the name and history of khat. Introductory paragraphs focus on presenting the geographic and historical context of khat. We articluated the gap by adding the following (in bold): "Other studies have shown the economic impact of khat as a single commodity [9,23–27], but less attention has been paid to understanding nuances of constraints and opportunities faced by vendors in a more complex economic ecosystem. **Our research fills this gap by focusing on the interplay between commodities within a local ecosystem.**" Also, right before the methods section, wording was added to highlight the significance of our approach: "This research addresses those gaps by investigating how khat trade, in comparison with vegetable trade, **providing a more comprehensive analysis of how khat relatively** impacts businesses income and local tax revenues."
**Reviewer #2**	Refining paragraph transitions and avoiding redundancy would improve readability, while briefly referencing the methodological approach would help orient the reader.	The manuscript has been thoroughly reviewed for grammar, flow, and clarity.
**Reviewer #2**	The introduction would benefit from a more explicit statement of the research gap and a brief mention of the methodological approach to better orient the reader.	We added an explanation of the methodology right after listing the hypotheses.

**Reviewer #2**	Overall, while the introduction is rich in content and well-cited, it requires editorial refinement to enhance clarity, coherence, and scholarly impact.	The manuscript has been thoroughly reviewed for grammar, flow, and clarity.
**Reviewer #2**	The Methods section would benefit from clearer organization, with labeled subsections to improve readability, and more consistent terminology.	The Methods section was significantly expanded and re-organized to address these concerns.
**Reviewer #2**	The use of COREQ is mentioned but not explained, and the rationale for sample size could be supported by a clearer discussion of saturation criteria.	The COREQ was explained in the revised Methods section.
**Reviewer #2**	Overall, the section is methodologically sound but could be refined for clarity and completeness.	The Methods section was significantly expanded and re-organized to address these concerns.
**Reviewer #2**	The Results section could be improved by simplifying repetitive narrative elements, clarifying the integration and interpretation of figures and tables, and refining the structure with clearer subheadings.	Subheadings were added; the narrative has been reviewed to simplify repetitiveness and clarify the use of tables and figures.
**Reviewer #2**	The contrast between khat and vegetable selling is compelling, especially in terms of profitability and barriers to entry, but would benefit from deeper qualitative insights and clearer explanation of cost structures.	Cost structures are presented in "Net annual income: Operating costs " section, in Figure 4 with explanation below. We discuss these findings extensively in that section and in the Discussion. For more detail, see S4 and S5 Tables.
**Reviewer #2**	Overall, the section is strong in content but would gain from editorial refinement and more effective data presentation.	The manuscript has been thoroughly reviewed for grammar, flow, and clarity. We have improved data presentation as well, with better titles, explanations of data, and references to all tables and figures in the text.
**Reviewer #2**	The Discussion and Recommendations section would benefit from clearer structure and deeper engagement with potential risks, such as over-reliance on khat, legal ambiguity, and food insecurity.	We have deepened the discussion of risks and recommendations in the Discussion section, addressing each of the topics mentionined in the review.

**Reviewer #2**	Expanding on how increased tax revenues could support public services and offering more concrete examples would strengthen the policy relevance of the recommendations.	The types of projects that could be funded with economic contribution of khat ristournes is first introduced in the section with that subheadding.
**Reviewer #2**	The Conclusion could be strengthened by suggesting directions for future research, such as longitudinal studies or comparative analyses, to deepen understanding and enhance generalizability.	At the end of the conclusion, we identified directions for future research.
**Reviewer #2**	Minor grammatical issues should be corrected throughout the manuscript. For instance, in the Introduction, “1 men” should be revised to “1 man”; “Sellers’type” to “Sellers’ type”; and “because of his legal uncertainty” likely refers to “because of its legal uncertainty.” These corrections should be applied consistently across all sections. A thorough grammar and style revision is recommended to ensure smoother flow and greater clarity.	The manuscript has been thoroughly reviewed for grammar, flow, and clarity. It is still possible that we have missed some clerical errors.
**Reviewer #3**	The authors have calculated Khat-related earnings from municipal taxation, but they don't report employment generation through its marketing.	Future research
**Reviewer #3**	Khat is not a substitute for human consumptions as a vegetable, thus comparison of Khat sellers with vegetable seller does not make sense.	We state in the introduction that we are studying khat and vegetables because of their complementary role in the economic ecosystem, both because they are both food. This was also reiterated at the beginning of the section "Economic outcomes of khat and vegetable trade"
**Reviewer #3**	Data presented in Table 1 indicate that most of the Khat sellers were women. Thus, it is suggested that women role in its marketing should be highlighted.	Future research
**Reviewer #3**	Heading of Table 1 should be 'Sample Details'.	Re-labeled.
**Reviewer #3**	In Table 2 sample size should be given i.e. (n=20).	This was added in the Gross annual income section.

**Reviewer #3**	Presenting data in Ar may be replaced with $, or alternatively in thousand or million Ar, without decimals.	Amounts have been adjusted. Annual amounts are presented in Million Ar but small amounts remain in Ar.
**Reviewer #3**	In figure 1 & 2 fiscal revenues have been given, which need to be reconsidered, as it is a more boarder term and includes non-tax revenues and grants etc. too.	We stated in the narrative the percentage of ristournes in all fiscal revenues of the municipalities.
**Reviewer #3**	On page 12/32, it is stated that ristournes have exponential growth, to clarify it, researchers are suggested to add trend lines.	The trend is shown in figs 1 and 2 in the histogram bar to avoid redundancy. Some explanation is provided on page 16 (on Economic impact of the khat trade Section), but language about 'exponential growth' has been deleted.
**Reviewer #3**	On page 13/32, whole data presented in Fig 3 and 4 has been represented in text. Thus, it is suggested either to delete figures, or to keep these with description of just main sources in the text.	We deleted the figures and added in the narrative the most important point: that ristournes is the highest fiscal revenue of the municipalities.
**Reviewer #3**	Table 2 contains annual income statement of all the sellers, instead of this, it is suggested to please present mean income of vendors along with STDs.	We have deleted the table (put it as Supporting Information Caption S2 and S2 Tables) and created Fig 6 to present this data. Standard deviation is described in the manuscript text.
**Reviewer #3**	On page 15/32, along with median income of vendors, their mean income should also be given.	We have presented this in fig 6: "Annual Income Variability".
**Reviewer #3**	In fig. 5, clearly indicate that it is monthly data.	No. It's annual data.
**Reviewer #3**	In fig. 6 & 7 purchase price per unit (e.g. bunch/ kg) and income per vendor should be given.	We have modified this in Table 2
**Reviewer #3**	Insert data in all the bar diagram for more clarity of readers.	We have modified this. See figures 3-8
**Reviewer #3**	In Table 6, add a row of sale volume for both Khat and Vegetables.	We have modified this. See table 2

**Reviewer #3**	It is stated in the article that vegetable markets are crowded. Vegetables are essential food consumption items, thus development of spacious market with related facilities should be suggested.	We added this recommendation at the end of the section "Relative advantages of khat and vegetable selling"

Editorial's comments

**Requirement**	**Observation/Comments from authors**
**1. PLOS ONE Style Requirements:** Ensure manuscript meets style requirements (formatting, file naming).	The manuscript files and content have been checked and revised to comply with the PLOS ONE style templates for the main body and title/affiliations pages.
**2. Inclusivity in Global Research Questionnaire:** Include a complete copy of PLOS' questionnaire on inclusivity in global research.	Supporting Information File: S1 Checklist added on Ethical clearance section
**3. Participant Consent Details:** Provide additional details regarding participant consent in the Methods section and online submission, specifically: (1) if consent was **informed** and (2) **what type** (written/verbal, how documented/witnessed). Address minors if applicable.	The methods section has been expanded to include this.

**4. Role of Funder Statement:** State what role the funders (University of Alabama at Birmingham) took in the study.	The funders had no role in study design, data collection and analysis, decision to publish, or preparation of the manuscript.
**5. Data Availability Statement (DAS):** Review the plan to make data available on acceptance; decide on a firm data sharing plan and revise the DAS if needed to explain any exemption reasoning if the open data policy cannot be adhered to.	Data Availability Statement (Online Submission Form).<br>We confirm we will make the fully anonymized data set freely accessible upon acceptance, in accordance with the Data Policy
**6. Figure References:** Ensure that Figure 3 and Figure 4 are referred to in the text of the manuscript.	All figures and tables are referred to in the text of the manuscript.
**7. Table References:** Ensure that Table 1 and Tables 4-6 are referred to in the text of the manuscript.	All figures and tables are referred to in the text of the manuscript.
**8. Supporting Information Captions:** Include captions for all Supporting Information files at the end of the manuscript and update in-text citations.	Captions for all Supporting Information files (including the newly added Inclusivity Questionnaire and the revised data file) have been added at the end of the main text. In-text citations have been checked.

**9. Identifying Data in Supporting Information:** Remove or anonymize all personal identifying data from the file "Minimal data_Kotra and Gezon_PlosOne.xlsx" and re-upload the fully anonymized dataset.	The data file has been thoroughly reviewed. All potentially identifying variables (e.g., detailed location data) have been removed, aggregated, or fully anonymized to ensure participant privacy and compliance with the Data Policy. The file will be re-uploaded.
**10. Specific Citations from Reviewers:** Review and evaluate publications recommended by reviewers to determine if they are relevant and should be cited (no strict requirement unless the editor indicated otherwise).	The recommended citation was added.
