# Supplementary material for: An assessment of the khat and vegetable trade in the local economic ecosystem: The case of northern Madagascar
Source: PLoS One. 2026 Jun 11;21(6):e0331722. doi: 10.1371/journal.pone.0331722 (PMC13257991; doi:10.1371/journal.pone.0331722)
Supplement: Attachment — Submitted filename: Response to reviewers.pdf [file pone.0331722.s010.pdf]

KOTRA Mahandrisoa Candicia,
candiciakotra@gmail.com
+393513699559/+261328267489
University of Antsiranana, Madagascar

February 14th, 2026

Subject: Submission of Revised Manuscript [PONE-D-25-38409R1] - [EMID:c67134201332a822]

Dear Editorial Committee,

Thank you for your second response dated January 22nd 2026 regarding our manuscript, "**An assessment of the khat and vegetable trade in the local economic ecosystem: The case of northern Madagascar" - PONE-D-25-38409R1,** which you have conditionally accepted pending minor revisions. We appreciate the time and effort the reviewers dedicated to evaluating our work; their insightful comments have been invaluable in strengthening the manuscript.

We have carefully considered all comments and suggestions provided by the reviewers. Our manuscript has been thoroughly revised according to their feedback. Our primary changes include :

- Clarifying the methodological approach in the Abstract and in the Methods section, enhancing presentation of qualitative data, as requested by the reviewer 4.
- Making data and methods public without restrictions on Harvard Dataverse and on protocol.io following PlOS One's guidelines.
- Updating the reference and bibliography following NLM Style Vancouver.

For your convenience, we have prepared a point-by-point response to every comment raised by Reviewers and editorial committee. This detailed response is provided in the appended document, where we indicate the specific location (section) of the changes in the revised manuscript.

We believe that these revisions have substantially improved the clarity, consistency, and scholarly impact of our article, making it suitable for publication in the PLOS One.

We look forward to hearing from you regarding the suitability of the second revised manuscript.

Sincerely,

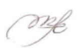

KOTRA Mahandrisoa Candicia

Comment	Location in Article and Comments
Provide Response to Reviewers letter, tracked-changes manuscript, and clean manuscript.	The manuscript was revised following reviewer's comments and submitted in track changes and in a clean manuscript.
Study population and design not shown in the title.	Done. The title is accurately revised and following PLOS one guidelines format. 110 characters
Question about using keywords before abstract; possible format issue.	Done. Keywords have been moved after the Abstract.
Methods part of abstract needs major revision: sampling strategies and data analysis must be explained.	Done
Why ethical clearance is at the beginning of methods?	We placed the ethical clearance statement at the beginning of the Methods section in order to explicitly address the Academic Editor's previous comments regarding the "Inclusivity in Global Research Questionnaire" and to clearly demonstrate that the study strictly followed PlOS One's ethical standards. This placement allows us to first clarify how participants were identified and contacted, how institutional authorization was obtained, and how informed consent was secured prior to participation. We considered it important to establish this ethical framework before presenting the details of the study design, data collection procedures, and analytical strategy, as these methodological steps were conducted within the boundaries of the approved ethical protocol.
COREQ mentioned but not consistently reflected in methods.	Done. See in Methods section on: "Research Team and reflexivity", "study design/Overall approach", "Study Sites and Participants ", "Participant consent and recruitment" and data collection and analysis.
Mixed methods claimed but qualitative and quantitative parts not explained; sample size 20 not justified.	The "why" of mixed-method is explained on "study design/overall approach". Later, qualitative and quantitative data are separately explained in the Data Collection and Data Analysis sections. We explain the qualitative methods specifically under Data Collection: "During their visits with sellers, the team conducted participant observation and interviewed them about their perceptions of profitability, constraints, and general trade conditions."

No information on sample size calculation/strategy and recruitment.	Sample size strategy is explained on " Study design/Study Sites and Participants " and "Data collection". We explain that participant recruitment was challenging because many sellers were hesitant to participate because of khat's legal uncertainty. In another hand, since we choose an ethnographic approach, we opted to study a small sample size with high quality, accurate data rather than including many sellers with unreliable data. Participants' rights are detailed under "Participant consent and recruitment".
Who did interviews/observations and how instruments were developed not described.	Done. Under section " Data Collection"
Qualitative analysis (thematic, triangulation) and credibility not explained with citations.	Done. Under section "Data analysis/Qualitative analysis". Citations to support our theory is on "Research Team and Reflexivity"
Include quotations from qualitative data in the "Results" section.	Done. Under Results section in "Economic impact of the khat trade/ Economic contribution of Khat Ristournes"; under "Khat's price fluctuations" and also in Discussions under "Governance and recommendations"
Discussion too broad; arguments not well focused (e.g., comparison of khat and vegetable trade).	The Discussion is organized into five key arguments, each addressing a specific analytical dimension of the results.<br><br>First, in the section "Khat and economic development," we focus on the contribution of khat taxation (ristournes) to municipal revenues and local development. This section discusses our Hypotheses H1 and H2A and situates our findings within the existing literature showing that khat constitutes a highly profitable activity and a key economic component of the local economic ecosystem in northern Madagascar.<br><br>Second, the comparison between khat and vegetable trade is treated in a dedicated section entitled "Relative advantages of khat and vegetable selling." This section demonstrates that khat selling is, on average, more profitable than vegetable selling, while also showing that, because of the risks and constraints associated with khat, some actors deliberately choose to remain in vegetable trading. This argument is then extended in the subsection "Economic concerns: Food security and livelihood diversification," where we emphasize that an over-reliance on khat production would be risky for the local economy and that

	strengthening vegetable production remains essential to ensure food security and livelihood resilience, even if it is less profitable.<br><br>Third, in the section “Governance and recommendations,” we address the issue of the legal uncertainty surrounding khat, which is also widely discussed in the literature. This section explicitly connects our results on municipal tax revenues and economic impact with the need to improve regulatory and governance frameworks, and it highlights the necessity for local authorities to balance economic development objectives with social considerations.<br><br>Fourth, in “Health and social concerns,” , linked with governance, we discuss the negative effects of khat, particularly on young people, both in terms of health (addiction) and social behavior (disengagement from work and education). This section is analytically linked to the governance discussion and supports our recommendation to strengthen regulation, especially to limit youth access to khat.<br><br>Finally, in “Environmental concerns,”, always linked with governance, we examine the environmental impacts of khat production, especially its high water consumption and its perceived role in local water shortages in Diego-Suarez. We also report the producers’ perspective, who attribute these problems partly to climate change. This divergence of interpretations reinforces our conclusion that clearer public policies and better regulation of khat production and trade are needed.<br><br>Overall, the structure of the Discussion follows a clear analytical progression from economic impact, to comparison of activities, to governance (including social and environmental implications).
First line contradictory; unnecessary references to figures.	References to fig 7 and 8 have been removed.<br>The revised opening sentence now clearly states that the study adopts a mixed-methods approach in which qualitative data are validated by quantitative findings.

in addition to summary statistics, the data points behind means, medians and variance measures should be available. If there are restrictions on publicly sharing data—e.g. participant privacy or use of data from a third party—those must be specified.	Done. It is on S6 Table like Supporting Information Captions. Add on Harvard Dataverse DOI: https://doi.org/10.7910/DVN/ZIWHDF<br><br>Also, Data Availability Statement added in the manuscript. Dataset has been deposited in a Harvard Dataverse and is accessible via DOI: https://doi.org/10.7910/DVN/ZIWHDF.<br><br>To ensure reproducibility, the full study protocol has been deposited in **protocols.io** and is independently citable via the following DOI:<br><br>https://www.protocols.io/private/3B44729B05CA11F1AAE40A58A9FEAC02.
If the reviewer comments include a recommendation to cite specific previously published works, please review and evaluate these publications to determine whether they are relevant and should be cited. There is no requirement to cite these works unless the editor has indicated otherwise.	We carefully reviewed the publication recommended by the reviewer. After evaluation, we found that the authors' arguments converge with the discussion developed in our manuscript. Accordingly, we have incorporated this reference into the revised version of the manuscript, as it strengthens and supports our argument.
**Authors may adhere to the journal formatting style and guidelines.**<br>**PLOS One format guidelines :**	
• File Size	All supporting files comply with this requirement: DOCX files are 15 KB, the PDF file is 203 KB, and the XLSX file is 18 KB.

• Figures NAAS format	All figures follow the NAAS format and have been deposited in Harvard Dataverse with the following DOI https://doi.org/10.7910/DVN/ZIWHDF
• File format	The manuscript is provided in DOCX format without any restrictions
• Font	A standard font and font size have been used throughout the manuscript
• Headings	Headings are limited to 3 hierarchical levels, in accordance with journal guidelines.
• Layout and spacing	The manuscript is double-spaced.
• Page and line numbers	Page numbers are placed at the bottom right (formatted as 1/39), and continuous line numbering has been applied throughout the manuscript.
• Language	Done. In english
• Other elements	• Figure captions are inserted immediately after the first paragraph in which each figure is cited. Figure files are uploaded separately in the PLOS ONE submission system and in Harvard Dataverse (https://doi.org/10.7910/DVN/ZIWHDF).<br>• Tables are inserted immediately after the first paragraph in which they are cited.<br>• Supporting information files are uploaded separately in the PLOS ONE submission system and in Harvard Dataverse (https://doi.org/10.7910/DVN/ZIWHDF).
Ensure references are complete, correct, and handle any retracted articles appropriately.	Formatted by Zotero. Using : NLM Style Vancouver : Citing Medicine 2nd edition (citation sequence, brackets), following the International Committee of Medical Journal Editors (ICMJE) sample reference format. All links were verified and are accurate.<br><br>A detailed summary of all reference verifications and corrections is provided below (Reference Change Summary). All citations were cross-checked against original sources, and corrections were made where misattribution or inaccuracies were identified.

Reference Change Summary

Sentence / Paragraph (as in manuscript)	Issue Identified	Action Taken

is known scientifically as Catha edulis [6–11]	Incorrect citation place	Lauerman (2012) and Patel (2019) removed
Yemeni dock workers during the French colonial period [2,13,15]	Incorrect reference	Weir (1985) removed after verification
earning the label such as "green gold" in Kenya and Madagascar [5,15]	Misattributed reference	Corrected to Gezon (2016)
Other studies have shown the economic impact of khat as a single commodity [6,19–21]	Incorrect references	Armstrong (2008) and Gebissa (2012) removed due to citation errors
The structure of this section follows the Consolidated Criteria for Reporting Qualitative Research (COREQ) [29]	New reference	Reference added
The involvement of locally based team members… facilitated communication, trust-building, and data quality[29,30]	New references	References added
Kotra conducted face-to-face interviews, while a research assistant took notes to reduce bias [29]	New reference	Reference added
The analytical approach followed Latzko-Toth's (2009) framework [32]	New reference	Reference added
This contributes to the widely held perception that khat prices are seasonal—higher in the dry season when production is lower and availability is limited [33]	New reference	Reference added
Many studies have shown that khat helps reduce poverty [34,35]	Incorrect reference	Corrected to Dessie and Kinlund (2008, p.209)

highlighting khat's critical economic role, especially in low-income contexts [36]	Incorrect reference	Corrected to Beckerleg (2008a, p.757)
Others like [17] document that khat contributes to enhanced quality of life among producers	Over-citation	Reduced to Lesourd (2013) only
Some [13] argue that the khat economy has led to improved living conditions	Over-citation	Limited to Gezon (2013, p.199)
contributing to employment and income in the countries where it is grown and sold [6,21,34].	Sentence changed	Sentence revised and new verified reference added
particularly in low-income countries like Kenya, Yemen, Ethiopia, and Madagascar [15,36,39].	Incorrect reference included	Luqman removed; remaining references verified
than rely on purchasing from Antananarivo […]	Incorrect citation place	Reference removed
Others disagree, warning that khat undermines crop diversity and increases food insecurity by encouraging monoculture [28,39].	Incorrect reference	Tefera (2009) replaced with Dawide et al. (2022)
To protect food security, scholars have recommended regulating khat's expansion while promoting the viability of crop diversification and alternative livelihoods [27]	Incorrect reference	Corrected to Feyisa and Aune (2003)
reduce dependence on a single product [35]	Incorrect reference	Corrected to Dessie and Kinlund (2008)

we recommend generating local fiscal policies that strengthen tax recovery and coordinate between regional and municipal tax agents [1,21]	Incorrect reference	Corrected to Gudata et al. (2019)
This tension calls for evidence-based, proactive watershed management policies and practices[1,39].	Incorrect reference	Corrected to Dawide et al. (2022)
These suggestions mirror those who advocate for public health strategies to mitigate overuse of khat in Ethiopia [27]	Incorrect reference	Corrected to Feyisa and Aune (2003)
and improved irrigation infrastructure, to strengthen rural economies while reducing dependence on khat […].	Incorrect citation place	Reference removed (citation error)
Banning khat in regions where it is culturally embedded is unlikely[46]	Incorrect reference	Corrected to Patel (2019)
