# Supplementary material for: An assessment of the khat and vegetable trade in the local economic ecosystem: The case of northern Madagascar
Source: PLoS One. 2026 Jun 11;21(6):e0331722. doi: 10.1371/journal.pone.0331722 (PMC13257991; doi:10.1371/journal.pone.0331722)
Supplement: Peer review history

# Peer review: Decision Letter 0

**DOI:** 10.1371/journal.pone.0331722.r001

**Authors:** Annesha Sil

3 Nov 2025

PONE-D-25-38409

Khat and the economic ecosystem in northern Madagascar.

PLOS ONE

Dear Dr. Kotra,

Thank you for submitting your manuscript to PLOS ONE. After careful consideration, we feel that it has merit but does not fully meet PLOS ONE’s publication criteria as it currently stands. Therefore, we invite you to submit a revised version of the manuscript that addresses the points raised during the review process.

Please submit your revised manuscript by Dec 15 2025 11:59PM. If you will need more time than this to complete your revisions, please reply to this message or contact the journal office at plosone@plos.org. When you’re ready to submit your revision, log on to https://www.editorialmanager.com/pone/ and select the ‘Submissions Needing Revision’ folder to locate your manuscript file.

Please include the following items when submitting your revised manuscript:

- A rebuttal letter that responds to each point raised by the academic editor and reviewer(s). You should upload this letter as a separate file labeled ‘Response to Reviewers’.
- A marked-up copy of your manuscript that highlights changes made to the original version. You should upload this as a separate file labeled ‘Revised Manuscript with Track Changes’.
- An unmarked version of your revised paper without tracked changes. You should upload this as a separate file labeled ‘Manuscript’.

If you would like to make changes to your financial disclosure, please include your updated statement in your cover letter. Guidelines for resubmitting your figure files are available below the reviewer comments at the end of this letter.

If applicable, we recommend that you deposit your laboratory protocols in protocols.io to enhance the reproducibility of your results. Protocols.io assigns your protocol its own identifier (DOI) so that it can be cited independently in the future. For instructions see: https://journals.plos.org/plosone/s/submission-guidelines#loc-laboratory-protocols. Additionally, PLOS ONE offers an option for publishing peer-reviewed Lab Protocol articles, which describe protocols hosted on protocols.io. Read more information on sharing protocols at https://plos.org/protocols?utm_medium=editorial-email&utm_source=authorletters&utm_campaign=protocols.

We look forward to receiving your revised manuscript.

Kind regards,

Annesha Sil, Ph.D.

Staff Editor

PLOS ONE

Journal Requirements:

When submitting your revision, we need you to address these additional requirements.

1.Please ensure that your manuscript meets PLOS ONE’s style requirements, including those for file naming. The PLOS ONE style templates can be found at

https://journals.plos.org/plosone/s/file?id=wjVg/PLOSOne_formatting_sample_main_body.pdf and

https://journals.plos.org/plosone/s/file?id=ba62/PLOSOne_formatting_sample_title_authors_affiliations.pdf

2. Please include a complete copy of PLOS’ questionnaire on inclusivity in global research in your revised manuscript. Our policy for research in this area aims to improve transparency in the reporting of research performed outside of researchers’ own country or community. The policy applies to researchers who have travelled to a different country to conduct research, research with Indigenous populations or their lands, and research on cultural artefacts. The questionnaire can also be requested at the journal’s discretion for any other submissions, even if these conditions are not met.  Please find more information on the policy and a link to download a blank copy of the questionnaire here: https://journals.plos.org/plosone/s/best-practices-in-research-reporting. Please upload a completed version of your questionnaire as Supporting Information when you resubmit your manuscript.

3. Please provide additional details regarding participant consent. In the ethics statement in the Methods and online submission information, please ensure that you have specified (1) whether consent was informed and (2) what type you obtained (for instance, written or verbal, and if verbal, how it was documented and witnessed). If your study included minors, state whether you obtained consent from parents or guardians. If the need for consent was waived by the ethics committee, please include this information.

If you are reporting a retrospective study of medical records or archived samples, please ensure that you have discussed whether all data were fully anonymized before you accessed them and/or whether the IRB or ethics committee waived the requirement for informed consent. If patients provided informed written consent to have data from their medical records used in research, please include this information.

4. Thank you for stating the following financial disclosure:

“The research was funded from University of Alabama at Birmingham, but not with a specific Grant so it doesn’t have a Grant number.

Initials of authors who received salary from commercial companies: Kotra.

URLs to sponsors’ websites : https://www.uab.edu/home/”

Please state what role the funders took in the study.  If the funders had no role, please state: "The funders had no role in study design, data collection and analysis, decision to publish, or preparation of the manuscript."

If this statement is not correct you must amend it as needed.

Please include this amended Role of Funder statement in your cover letter; we will change the online submission form on your behalf.

5. When completing the data availability statement of the submission form, you indicated that you will make your data available on acceptance. We strongly recommend all authors decide on a data sharing plan before acceptance, as the process can be lengthy and hold up publication timelines. Please note that, though access restrictions are acceptable now, your entire data will need to be made freely accessible if your manuscript is accepted for publication. This policy applies to all data except where public deposition would breach compliance with the protocol approved by your research ethics board. If you are unable to adhere to our open data policy, please kindly revise your statement to explain your reasoning and we will seek the editor’s input on an exemption. Please be assured that, once you have provided your new statement, the assessment of your exemption will not hold up the peer review process.

6. Please ensure that you refer to Figure 3 and 4 in your text as, if accepted, production will need this reference to link the reader to the figure.

7. We note you have included a table to which you do not refer in the text of your manuscript. Please ensure that you refer to Table 1 and 4-6 in your text; if accepted, production will need this reference to link the reader to the Table.

8. Please include captions for your Supporting Information files at the end of your manuscript, and update any in-text citations to match accordingly. Please see our Supporting Information guidelines for more information: http://journals.plos.org/plosone/s/supporting-information.

9. We note that there is identifying data in the Supporting Information file “Minimal data_Kotra and Gezon_PlosOne.xlsx”. Due to the inclusion of these potentially identifying data, we have removed this file from your file inventory. Prior to sharing human research participant data, authors should consult with an ethics committee to ensure data are shared in accordance with participant consent and all applicable local laws.

Data sharing should never compromise participant privacy. It is therefore not appropriate to publicly share personally identifiable data on human research participants. The following are examples of data that should not be shared:

-Name, initials, physical address

-Ages more specific than whole numbers

-Internet protocol (IP) address

-Specific dates (birth dates, death dates, examination dates, etc.)

-Contact information such as phone number or email address

-Location data

-ID numbers that seem specific (long numbers, include initials, titled “Hospital ID”) rather than random (small numbers in numerical order)

Data that are not directly identifying may also be inappropriate to share, as in combination they can become identifying. For example, data collected from a small group of participants, vulnerable populations, or private groups should not be shared if they involve indirect identifiers (such as sex, ethnicity, location, etc.) that may risk the identification of study participants.

Additional guidance on preparing raw data for publication can be found in our Data Policy (https://journals.plos.org/plosone/s/data-availability#loc-human-research-participant-data-and-other-sensitive-data) and in the following article: http://www.bmj.com/content/340/bmj.c181.long.

Please remove or anonymize all personal information (<specific identifying information in file to be removed>), ensure that the data shared are in accordance with participant consent, and re-upload a fully anonymized data set. Please note that spreadsheet columns with personal information must be removed and not hidden as all hidden columns will appear in the published file.

10. If the reviewer comments include a recommendation to cite specific previously published works, please review and evaluate these publications to determine whether they are relevant and should be cited. There is no requirement to cite these works unless the editor has indicated otherwise.

[Note: HTML markup is below. Please do not edit.]

Reviewers’ comments:

Reviewer’s Responses to Questions

**Comments to the Author**

1. Is the manuscript technically sound, and do the data support the conclusions?

The manuscript must describe a technically sound piece of scientific research with data that supports the conclusions. Experiments must have been conducted rigorously, with appropriate controls, replication, and sample sizes. The conclusions must be drawn appropriately based on the data presented.

Reviewer #1: Yes

Reviewer #2: Yes

Reviewer #3: Yes

**********

2. Has the statistical analysis been performed appropriately and rigorously?

Reviewer #1: Yes

Reviewer #2: Yes

Reviewer #3: N/A

**********

3. Have the authors made all data underlying the findings in their manuscript fully available?

The PLOS Data policy requires authors to make all data underlying the findings described in their manuscript fully available without restriction, with rare exception (please refer to the Data Availability Statement in the manuscript PDF file). The data should be provided as part of the manuscript or its supporting information, or deposited to a public repository. For example, in addition to summary statistics, the data points behind means, medians and variance measures should be available. If there are restrictions on publicly sharing data—e.g. participant privacy or use of data from a third party—those must be specified.

Reviewer #1: Yes

Reviewer #2: Yes

Reviewer #3: Yes

**********

4. Is the manuscript presented in an intelligible fashion and written in standard English?

PLOS ONE does not copyedit accepted manuscripts, so the language in submitted articles must be clear, correct, and unambiguous. Any typographical or grammatical errors should be corrected at revision, so please note any specific errors here.

Reviewer #1: Yes

Reviewer #2: Yes

Reviewer #3: Yes

**********

5. Review Comments to the Author

Please use the space provided to explain your answers to the questions above. You may also include additional comments for the author, including concerns about dual publication, research ethics, or publication ethics. (Please upload your review as an attachment if it exceeds 20,000 characters)

Reviewer #1: Authors should provide more details on the mixed-methods approach beyond just qualitative and quantitative methods in the abstract. Clarify the different scientific names for Catha edulis (Forssk, Forsk, Forskal) used in the introduction for consistency. Elaborate on the dynamic complementarity between khat and vegetable farming to provide a clearer understanding of their intertwined roles in the economic ecosystem.

Provide more context or a brief explanation for the use of cross-dynamic tables in data analysis, as it might not be a universally familiar term. When discussing the exponential growth trend of khat ristournes collection, provide a brief explanation of what ristournes are for readers unfamiliar with the term.

Ensure consistency in the description of ristournes as both "rebates and tax levies" or just "tax levies" throughout the text for clarity. Strengthen the discussion on how the study’s findings specifically confirm previous research, rather than just stating it generally. All the figures must be written with clear labeling and captions instead of giving a title in the figure itself.

The authors should offer more detailed examples or mechanisms that demonstrate how local fiscal policies can enhance tax recovery and coordination. Elaborate on the proposed regulated legality for khat, outlining what specific regulations may include addressing public health and harm reduction.

Consider exploring the potential for collaborative marketing strategies, both locally and globally, to further legitimize the khat industry, enhance its economic benefits, and potentially expand its reach, while adhering to regulatory frameworks similar to those for other legal psychoactive crops like tobacco, tea, and coffee. (Refer to the paper "Collaborative Marketing Strategies in Agriculture for Global Reach and Local Impact." Emerging Trends in Food and Agribusiness Marketing, edited by Satish Chandra Pant, et al., IGI Global Scientific Publishing, 2025, pp. 219-252. https://doi.org/10.4018/979-8-3693-6715-5.ch008)

Reviewer #2: The Introduction presents a well-researched overview of khat’s botanical identity, cultural relevance, and economic role, especially in Madagascar. It effectively transitions from global context to local focus and includes relevant legal and governance aspects. However, the section would benefit from clearer structure, more concise language, and a stronger articulation of the research gap. Refining paragraph transitions and avoiding redundancy would improve readability, while briefly referencing the methodological approach would help orient the reader. The introduction would benefit from a more explicit statement of the research gap and a brief mention of the methodological approach to better orient the reader. Overall, while the introduction is rich in content and well-cited, it requires editorial refinement to enhance clarity, coherence, and scholarly impact.

The Methods section presents a thorough and context-aware account of the study’s design, including ethical approvals, sampling strategies, data collection, and analysis. It reflects a careful approach to fieldwork in a legally and socially sensitive environment, combining qualitative and quantitative methods appropriately. The authors acknowledge recruitment challenges and describe efforts to build trust with participants, which strengthens the credibility of the data. However, the section would benefit from clearer organization, with labeled subsections to improve readability, and more consistent terminology. The use of COREQ is mentioned but not explained, and the rationale for sample size could be supported by a clearer discussion of saturation criteria. Overall, the section is methodologically sound but could be refined for clarity and completeness.

The Results section presents a detailed and well-organized analysis of khat’s economic role in northern Madagascar, particularly its contribution to municipal tax revenue and vendor income. The findings are clearly linked to the study’s hypothesis and supported by rich fiscal data and seasonal comparisons. However, the section could be improved by simplifying repetitive narrative elements, clarifying the integration and interpretation of figures and tables, and refining the structure with clearer subheadings. The contrast between khat and vegetable selling is compelling, especially in terms of profitability and barriers to entry, but would benefit from deeper qualitative insights and clearer explanation of cost structures. Overall, the section is strong in content but would gain from editorial refinement and more effective data presentation.

The Discussion and Recommendations section offers a well-rounded synthesis of the study’s findings, effectively linking them to existing literature and broader development goals. It confirms the central role of khat in local economic development and explores its complementary relationship with vegetable selling. The authors address health, social, environmental, and food security concerns with nuance, proposing regulation over prohibition and aligning their recommendations with Sustainable Development Goal 1. While the section is grounded in empirical evidence and avoids simplistic conclusions, it would benefit from clearer structure and deeper engagement with potential risks, such as over-reliance on khat, legal ambiguity, and food insecurity. Expanding on how increased tax revenues could support public services and offering more concrete examples would strengthen the policy relevance of the recommendations.

The Conclusion provides a concise and coherent summary of the study’s main contributions, emphasizing the value of a micro-level, ethnographic approach to understanding the khat trade in northern Madagascar. It reinforces the finding that khat plays a complementary role in the local economy and highlights barriers to entry for different types of vendors. Limitations such as the small sample size and the one-year data collection period are acknowledged with transparency. While the section is well-aligned with the rest of the manuscript, it could be strengthened by suggesting directions for future research, such as longitudinal studies or comparative analyses, to deepen understanding and enhance generalizability.

Minor grammatical issues should be corrected throughout the manuscript. For instance, in the Introduction, “1 men” should be revised to “1 man”; “Sellers’type” to “Sellers’ type”; and “because of his legal uncertainty” likely refers to “because of its legal uncertainty.” These corrections should be applied consistently across all sections. A thorough grammar and style revision is recommended to ensure smoother flow and greater clarity.

Reviewer #3: The article is acceptable for publishing with minor modifications. The authors have calculated Khat-related earnings from municipal taxation, but they don’t report employment generation through its marketing. Khat is not a substitute for human consumptions as a vegetable, thus comparison of Khat sellers with vegetable seller does not make sense. Data presented in Table 1 indicate that most of the Khat sellers were women. Thus, it is suggested that women role in its marketing should be highlighted. Heading of Table 1 should be ‘Sample Details’. In Table 2 sample size should be given i.e. (n=20). Presenting data in Ar may be replaced with $, or alternatively in thousand or million Ar, without decimals. In figure 1 & 2 fiscal revenues have been given, which need to be reconsidered, as it is a more boarder term and includes non-tax revenues and grants etc. too. On page 12/32, it is stated that ristournes have exponential growth, to clarify it, researchers are suggested to add trend lines. On page 13/32, whole data presented in Fig 3 and 4 has been represented in text. Thus, it is suggested either to delete figures, or to keep these with description of just main sources in the text. Table 2 contains annual income statement of all the sellers, instead of this, it is suggested to please present mean income of vendors along with STDs. On page 15/32, along with median income of vendors, their mean income should also be given. In fig. 5, clearly indicate that it is monthly data. In fig. 6 & 7 purchase price per unit (e.g. bunch/ kg) and income per vendor should be given. Insert data in all the bar diagram for more clarity of readers. In Table 6, add a row of sale volume for both Khat and Vegetables. It is stated in the article that vegetable markets are crowded. Vegetables are essential food consumption items, thus development of spacious market with related facilities should be suggested.

**********

6. PLOS authors have the option to publish the peer review history of their article (what does this mean?). If published, this will include your full peer review and any attached files.

If you choose “no”, your identity will remain anonymous but your review may still be made public.

**Do you want your identity to be public for this peer review?**  For information about this choice, including consent withdrawal, please see our Privacy Policy.

Reviewer #1: No

Reviewer #2: No

Reviewer #3: No

**********

[NOTE: If reviewer comments were submitted as an attachment file, they will be attached to this email and accessible via the submission site. Please log into your account, locate the manuscript record, and check for the action link "View Attachments". If this link does not appear, there are no attachment files.]

To ensure your figures meet our technical requirements, please review our figure guidelines: https://journals.plos.org/plosone/s/figures

You may also use PLOS’s free figure tool, NAAS, to help you prepare publication quality figures: https://journals.plos.org/plosone/s/figures#loc-tools-for-figure-preparation.

NAAS will assess whether your figures meet our technical requirements by comparing each figure against our figure specifications.

Attachment Submitted filename: PLOS ONE_paper revision_22_10_2025.pdf

# Author comment: Author response to Decision Letter 1

**DOI:** 10.1371/journal.pone.0331722.r002

15 Dec 2025

Reviewer 3: In fig. 5, clearly indicate that it is monthly data --> It is annual data (our calculations are annual).

Attachment Submitted filename: Response to reviewers_khat paper_Kotra and Gezon.pdf

# Peer review: Decision Letter 1

**DOI:** 10.1371/journal.pone.0331722.r003

**Authors:** Mohammed Badeso

22 Jan 2026

PONE-D-25-38409R1

Khat and the economic ecosystem in northern Madagascar.

PLOS One

Dear Dr. Kotra,

Thank you for submitting your manuscript to PLOS ONE. After careful consideration, we feel that it has merit but does not fully meet PLOS ONE’s publication criteria as it currently stands. Therefore, we invite you to submit a revised version of the manuscript that addresses the points raised during the review process.

Please submit your revised manuscript by Mar 08 2026 11:59PM. If you will need more time than this to complete your revisions, please reply to this message or contact the journal office at plosone@plos.org. When you’re ready to submit your revision, log on to https://www.editorialmanager.com/pone/ and select the ‘Submissions Needing Revision’ folder to locate your manuscript file.

Please include the following items when submitting your revised manuscript:

- A letter that responds to each point raised by the academic editor and reviewer(s). You should upload this letter as a separate file labeled ‘Response to Reviewers’.
- A marked-up copy of your manuscript that highlights changes made to the original version. You should upload this as a separate file labeled ‘Revised Manuscript with Track Changes’.
- An unmarked version of your revised paper without tracked changes. You should upload this as a separate file labeled ‘Manuscript’.

-->If you would like to make changes to your financial disclosure, please include your updated statement in your cover letter. Guidelines for resubmitting your figure files are available below the reviewer comments at the end of this letter.

If applicable, we recommend that you deposit your laboratory protocols in protocols.io to enhance the reproducibility of your results. Protocols.io assigns your protocol its own identifier (DOI) so that it can be cited independently in the future. For instructions see: https://journals.plos.org/plosone/s/submission-guidelines#loc-laboratory-protocols. Additionally, PLOS ONE offers an option for publishing peer-reviewed Lab Protocol articles, which describe protocols hosted on protocols.io. Read more information on sharing protocols at https://plos.org/protocols?utm_medium=editorial-email&utm_source=authorletters&utm_campaign=protocols.

We look forward to receiving your revised manuscript.

Kind regards,

Mohammed Hasen Badeso, Epidemiologist

Academic Editor

PLOS One

Journal Requirements:

If the reviewer comments include a recommendation to cite specific previously published works, please review and evaluate these publications to determine whether they are relevant and should be cited. There is no requirement to cite these works unless the editor has indicated otherwise.

Please review your reference list to ensure that it is complete and correct. If you have cited papers that have been retracted, please include the rationale for doing so in the manuscript text, or remove these references and replace them with relevant current references. Any changes to the reference list should be mentioned in the rebuttal letter that accompanies your revised manuscript. If you need to cite a retracted article, indicate the article’s retracted status in the References list and also include a citation and full reference for the retraction notice.

[Note: HTML markup is below. Please do not edit.]

Reviewers’ comments:

Reviewer’s Responses to Questions

**Comments to the Author**

1. If the authors have adequately addressed your comments raised in a previous round of review and you feel that this manuscript is now acceptable for publication, you may indicate that here to bypass the “Comments to the Author” section, enter your conflict of interest statement in the “Confidential to Editor” section, and submit your "Accept" recommendation.

Reviewer #1: All comments have been addressed

Reviewer #4: (No Response)

**********

2. Is the manuscript technically sound, and do the data support the conclusions?

The manuscript must describe a technically sound piece of scientific research with data that supports the conclusions. Experiments must have been conducted rigorously, with appropriate controls, replication, and sample sizes. The conclusions must be drawn appropriately based on the data presented.

Reviewer #1: Yes

Reviewer #4: Yes

**********

3. Has the statistical analysis been performed appropriately and rigorously?

Reviewer #1: Yes

Reviewer #4: No

**********

4. Have the authors made all data underlying the findings in their manuscript fully available?

The PLOS Data policy requires authors to make all data underlying the findings described in their manuscript fully available without restriction, with rare exception (please refer to the Data Availability Statement in the manuscript PDF file). The data should be provided as part of the manuscript or its supporting information, or deposited to a public repository. For example, in addition to summary statistics, the data points behind means, medians and variance measures should be available. If there are restrictions on publicly sharing data—e.g. participant privacy or use of data from a third party—those must be specified.

Reviewer #1: Yes

Reviewer #4: Yes

**********

5. Is the manuscript presented in an intelligible fashion and written in standard English?

PLOS ONE does not copyedit accepted manuscripts, so the language in submitted articles must be clear, correct, and unambiguous. Any typographical or grammatical errors should be corrected at revision, so please note any specific errors here.

Reviewer #1: Yes

Reviewer #4: Yes

**********

6. Review Comments to the Author

Please use the space provided to explain your answers to the questions above. You may also include additional comments for the author, including concerns about dual publication, research ethics, or publication ethics. (Please upload your review as an attachment if it exceeds 20,000 characters)

Reviewer #1: Authors have incorporated all of my suggestions, and the paper quality is enhanced. Authors may adhere to the journal formatting style and guidelines.

Reviewer #4: Journal Name: PLOS One

Manuscript number: PONE-D-25-38409R1

Manuscript title: Khat and the economic ecosystem in northern Madagascar.

To editor,

This manuscript is very interesting and exploring the public health concerns that is under studied. The manuscript is publishable. However, I would like to offer the following comments and suggestions:

1.Title: Study population and design is not showing in the title. Need to revise.

2.Keywords: I don’t know about the using keywords before writing abstract? Is it journal format issue?

3.Abstract

Method section need to revise grossly. Sampling strategies and data analysis method need to explain. Please revise.

4.Introduction

Introduction section is very okay

5.Methods

Methods section is need to gross revisions.

-Why the author writes the ethical clearance part at the very beginning of the methods section?

-In methods section, the author well explained about COREQ but its reflections not well described in the entire methods section (effort to maximize the items).

-For mixed method study, the author just named it but qualitative and quantitative methods not explained. Data collected in two phases and sample size is only 20 is not justifiable? Quantitative sample size, sampling strategies are not explained.

- No information provided about the sample size selection strategy and justification. Because, how the study participants recruited, participants’ rights, interview guidelines were developed did not describe. Who conducted observation, interviews and other responsibilities is not explained. Better to revise it into separate part like sample size and sampling strategies and then data collection technique and procedures. Additionally, it would be more evident if the authors follow the COREQ as the authors mentioned about its use.

-Data analysis section is not consistent. What about qualitative data like thematic synthesis, triangulation process need to explain clearly with citation. How the credibility ensures need to explain with citation?

6.Results

I would ask to include the quotations from the qualitative data.

7.Discussion

- Too broad and the arguments of this paper is not well presented like comparison of Khat and vegetable trade.

8.Conclusion

-First line is contradictory. Mixed-methods, ethnographically…. Why the authors refers for fig. 7, 8 with details in conclusion?

With thanks

Md. Shahgahan Miah, Ph.D.

**********

7. PLOS authors have the option to publish the peer review history of their article (what does this mean?). If published, this will include your full peer review and any attached files.

If you choose “no”, your identity will remain anonymous but your review may still be made public.

**Do you want your identity to be public for this peer review?**  For information about this choice, including consent withdrawal, please see our Privacy Policy.

Reviewer #1: No

Reviewer #4: **Yes:**  Dr. Md. Shahgahan Miah

**********

[NOTE: If reviewer comments were submitted as an attachment file, they will be attached to this email and accessible via the submission site. Please log into your account, locate the manuscript record, and check for the action link "View Attachments". If this link does not appear, there are no attachment files.]

To ensure your figures meet our technical requirements, please review our figure guidelines: https://journals.plos.org/plosone/s/figures

You may also use PLOS’s free figure tool, NAAS, to help you prepare publication quality figures: https://journals.plos.org/plosone/s/figures#loc-tools-for-figure-preparation.

NAAS will assess whether your figures meet our technical requirements by comparing each figure against our figure specifications.

# Author comment: Author response to Decision Letter 2

**DOI:** 10.1371/journal.pone.0331722.r004

14 Feb 2026

Dear Editorial Committee,

We acknowledge the importance of strict adherence to PLOS ONE formatting guidelines, and we appreciate the opportunity to clarify our approach.

1. We carefully reviewed the publication recommended by the reviewer. After evaluation, we found that the authors’ arguments converge with the discussion developed in our manuscript. Accordingly, we have incorporated this reference into the revised version of the manuscript, as it strengthens and supports our argument.

2. All data have been deposited in a public repository without restriction, in accordance with your recommendation.

3. All references have been carefully checked and corrected. We apologize for any inconsistencies in the previous version of the manuscript.

Thank you for your consideration.

Kind regards,

Attachment Submitted filename: Response to reviewers.pdf

# Peer review: Decision Letter 2

**DOI:** 10.1371/journal.pone.0331722.r005

**Authors:** Miquel Vall-llosera Camps

17 Apr 2026

An assessment of the khat and vegetable trade in the local economic ecosystem: The case of northern Madagascar

PONE-D-25-38409R2

Dear Dr. Kotra,

We’re pleased to inform you that your manuscript has been judged scientifically suitable for publication and will be formally accepted for publication once it meets all outstanding technical requirements.

Within one week, you’ll receive an e-mail detailing the required amendments. When these have been addressed, you’ll receive a formal acceptance letter and your manuscript will be scheduled for publication.

An invoice will be generated when your article is formally accepted. Please note, if your institution has a publishing partnership with PLOS and your article meets the relevant criteria, all or part of your publication costs will be covered. Please make sure your user information is up-to-date by logging into Editorial Manager at Editorial Manager® and clicking the ‘Update My Information’ link at the top of the page. For questions related to billing, please contact billing support.

If your institution or institutions have a press office, please notify them about your upcoming paper to help maximize its impact. If they’ll be preparing press materials, please inform our press team as soon as possible -- no later than 48 hours after receiving the formal acceptance. Your manuscript will remain under strict press embargo until 2 pm Eastern Time on the date of publication. For more information, please contact onepress@plos.org.

Kind regards,

Miquel Vall-llosera Camps

Senior Staff Editor

PLOS One

Reviewers’ comments:

Reviewer’s Responses to Questions

**Comments to the Author**

1. If the authors have adequately addressed your comments raised in a previous round of review and you feel that this manuscript is now acceptable for publication, you may indicate that here to bypass the “Comments to the Author” section, enter your conflict of interest statement in the “Confidential to Editor” section, and submit your "Accept" recommendation.

Reviewer #1: All comments have been addressed

**********

2. Is the manuscript technically sound, and do the data support the conclusions?

The manuscript must describe a technically sound piece of scientific research with data that supports the conclusions. Experiments must have been conducted rigorously, with appropriate controls, replication, and sample sizes. The conclusions must be drawn appropriately based on the data presented.

Reviewer #1: Yes

**********

3. Has the statistical analysis been performed appropriately and rigorously?

Reviewer #1: N/A

**********

4. Have the authors made all data underlying the findings in their manuscript fully available?

The PLOS Data policy requires authors to make all data underlying the findings described in their manuscript fully available without restriction, with rare exception (please refer to the Data Availability Statement in the manuscript PDF file). The data should be provided as part of the manuscript or its supporting information, or deposited to a public repository. For example, in addition to summary statistics, the data points behind means, medians and variance measures should be available. If there are restrictions on publicly sharing data—e.g. participant privacy or use of data from a third party—those must be specified.

Reviewer #1: Yes

**********

5. Is the manuscript presented in an intelligible fashion and written in standard English?

PLOS ONE does not copyedit accepted manuscripts, so the language in submitted articles must be clear, correct, and unambiguous. Any typographical or grammatical errors should be corrected at revision, so please note any specific errors here.

Reviewer #1: Yes

**********

6. Review Comments to the Author

Please use the space provided to explain your answers to the questions above. You may also include additional comments for the author, including concerns about dual publication, research ethics, or publication ethics. (Please upload your review as an attachment if it exceeds 20,000 characters)

Reviewer #1: The authors have incorporated all necessary corrections as per the suggestions. The paper may be considered for acceptance.

**********

7. PLOS authors have the option to publish the peer review history of their article (what does this mean?). If published, this will include your full peer review and any attached files.

If you choose “no”, your identity will remain anonymous but your review may still be made public.

**Do you want your identity to be public for this peer review?**  For information about this choice, including consent withdrawal, please see our Privacy Policy.

Reviewer #1: No

**********

# Editor report: Acceptance letter

**DOI:** 10.1371/journal.pone.0331722.r006

**Authors:** Miquel Vall-llosera Camps

PONE-D-25-38409R2

PLOS One

Dear Dr. Kotra,

I’m pleased to inform you that your manuscript has been deemed suitable for publication in PLOS One. Congratulations! Your manuscript is now being handed over to our production team.

At this stage, our production department will prepare your paper for publication. This includes ensuring the following:

* All references, tables, and figures are properly cited

* All relevant supporting information is included in the manuscript submission,

* There are no issues that prevent the paper from being properly typeset

You will receive further instructions from the production team, including instructions on how to review your proof when it is ready. Please keep in mind that we are working through a large volume of accepted articles, so please give us a few days to review your paper and let you know the next and final steps.

Lastly, if your institution or institutions have a press office, please let them know about your upcoming paper now to help maximize its impact. If they’ll be preparing press materials, please inform our press team within the next 48 hours. Your manuscript will remain under strict press embargo until 2 pm Eastern Time on the date of publication. For more information, please contact onepress@plos.org.

You will receive an invoice from PLOS for your publication fee after your manuscript has reached the completed accept phase. If you receive an email requesting payment before acceptance or for any other service, this may be a phishing scheme. Learn how to identify phishing emails and protect your accounts at https://explore.plos.org/phishing.

If we can help with anything else, please email us at customercare@plos.org.

Thank you for submitting your work to PLOS ONE and supporting open access.

Kind regards,

PLOS ONE Editorial Office Staff

on behalf of

Dr. Miquel Vall-llosera Camps

Staff Editor

PLOS One
